# Identification of Novel Genes in Human Airway Epithelial Cells associated with Chronic Obstructive Pulmonary Disease (COPD) using Machine-Based Learning Algorithms

**DOI:** 10.1038/s41598-018-33986-8

**Published:** 2018-10-25

**Authors:** Shayan Mostafaei, Anoshirvan Kazemnejad, Sadegh Azimzadeh Jamalkandi, Soroush Amirhashchi, Seamas C. Donnelly, Michelle E. Armstrong, Mohammad Doroudian

**Affiliations:** 1 Department of Biostatistics, Faculty of Medical Sciences, Tarbiat Modares University, Tehran, Iran; 2 Chemical Injuries Research Center, Systems Biology and Poisonings Institute, Baqiyatallah University of Medical Sciences, Tehran, Iran; 3 Department of Actuarial Science, Faculty of Mathematical Science, Shahid Beheshti University, Tehran, Iran; 4 Department of Clinical Medicine, School of Medicine, Trinity Biomedical Sciences Institute, Trinity College Dublin, Dublin 2, Ireland; 5 Department of Clinical Medicine, Trinity Centre for Health Sciences, Tallaght University Hospital, Tallaght, Dublin 24, Ireland

## Abstract

The aim of this project was to identify candidate novel therapeutic targets to facilitate the treatment of COPD using machine-based learning (ML) algorithms and penalized regression models. In this study, 59 healthy smokers, 53 healthy non-smokers and 21 COPD smokers (9 GOLD stage I and 12 GOLD stage II) were included (n = 133). 20,097 probes were generated from a small airway epithelium (SAE) microarray dataset obtained from these subjects previously. Subsequently, the association between gene expression levels and smoking and COPD, respectively, was assessed using: AdaBoost Classification Trees, Decision Tree, Gradient Boosting Machines, Naive Bayes, Neural Network, Random Forest, Support Vector Machine and adaptive LASSO, Elastic-Net, and Ridge logistic regression analyses. Using this methodology, we identified 44 candidate genes, 27 of these genes had been previously been reported as important factors in the pathogenesis of COPD or regulation of lung function. Here, we also identified 17 genes, which have not been previously identified to be associated with the pathogenesis of COPD or the regulation of lung function. The most significantly regulated of these genes included: PRKAR2B, GAD1, LINC00930 and SLITRK6. These novel genes may provide the basis for the future development of novel therapeutics in COPD and its associated morbidities.

## Introduction

Chronic obstructive pulmonary disease (COPD) is a progressive inflammatory disease characterized by airway obstruction and is predicted to be among the first three causes of death worldwide^1,2^. Clinical presentations include emphysema, small airway obstructions and chronic bronchitis. COPD has been shown to develop in 30% of smokers and smoking history, combined with reduced daily physical activity, may be the main risk factor associated with the development of COPD^3^. Additional risk factors in COPD, in genetically susceptible individuals, include a history of maternal smoking, second hand smoke, polluted air, maternal/paternal asthma, childhood asthma or respiratory infections and malnutrition^4^. Although COPD archetypically manifests itself in males, recent studies have demonstrated an increased incidence and mortality rates in females. Furthermore, female patients with COPD are more often misdiagnosed and/or underdiagnosed^5,6^.

From a genetic perspective, COPD is a complex disease arising from mutations in multiple alleles and the lack of integration of data in this disease has been attributed to dispersed, independent genome-wide association studies (GWAS)^7^. DNA microarrays now permit scientists to screen thousands of genes simultaneously in order to determine which genes are active, hyperactive or silent in normal or COPD tissue. Furthermore, network-based medicine has also been recently employed to facilitate the investigation of genomics, transcriptomics, proteomics and other “–omics” in order to better understand complex diseases, such as COPD^8^. However, from a biological perspective, only a only a small subset of genes identified by these methodologies will be strongly indicative of the target disease^9^. Therefore, in this study, we employed a novel methodology, namely machine-based learning algorithms combined with penalized regression models, in order to study genomic change in COPD in a more selective manner. Furthermore, we have also had a longstanding interest in the genetics of COPD, formally as part of a European Union consortium^10–13^. Here, we now extend on these initial observations.

This study was designed to apply signaling-network methodology with machine-based learning methods to better understand the genetic etiology of smoking exposure and COPD in 59 healthy smokers, 53 healthy non-smokers and 21 COPD smokers (9 of GOLD stage I and 12 of GOLD stage II) were included (Total: n = 133). Furthermore, AdaBoost Classification Trees, Decision Tree, Gradient Boosting Machines, Naive Bayes, Neural Network, Random Forest, Support Vector Machine (as machine learning algorithms) and adaptive LASSO, elastic-net, and ridge logistic regression (as statistical models) were also applied.

In summary, we identified 44 candidate genes associating with smoking exposure and the incidence/progression of COPD. We also identified 17 novel genes, which were not previously associated with COPD, the regulation of lung function or smoking exposure. The most significantly regulated of these genes included: PRKAR2B, GAD1, LINC00930, and SLITRK6. These novel genes may provide the basis for the future development of novel therapeutics in COPD and warrant further investigation and validation.

## Results

### Differential analysis of gene expression data

**Figure 1:**
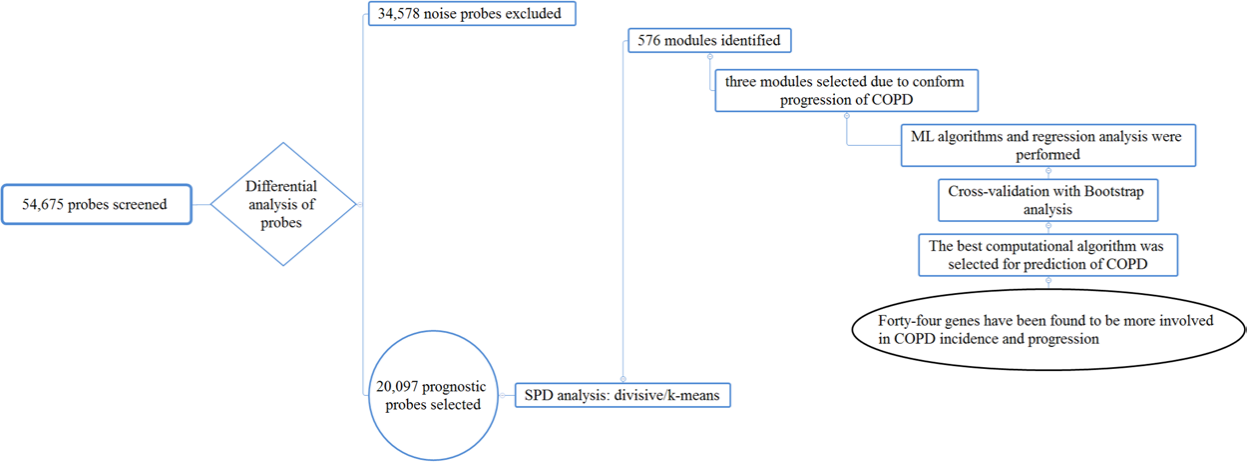
Schematic demonstrating study plan and flowchart.

In this study, 54,675 probes were screened using the microarray dataset generated from SAE cells previously from: 59 healthy smokers, 53 healthy non-smokers and 21 COPD smokers (42.8% of GOLD stage I and 57.2% of GOLD stage II) (Table 1)^14^. Differential analysis was subsequently performed in order to select 20,097 probes. Subsequently, 718 probes and 544 genes (Fig. 1) were identified which were significantly changed (all *p* values < 0.0001) in COPD patients compared with healthy non-smokers. These genes, which include USP27X, PPP4R4, AHRR, PRKAR2B, GAD1, CYP1A1 and CYP1B1, are listed in the Supplementary File S1.

**Table 1:** Basic characteristics of the study samples.

Characteristics		COPD Smoker (N = 21)	Healthy Smoker (N = 59)	Healthy Non-smoker (N = 53)	P-value
Age (Year)*		50.38 ± 7.081	42.93 ± 7.267	41.0 ± 11.30	<0.001
Smoking (pack per year)*		36.98 ± 23.953	27.6 ± 16.975	—	0.078
FVC*		97 ± 20	109 ± 13	107 ± 13	0.004
FEV1*		74 ± 20	107 ± 14	105 ± 14	<0.001
FEV1/FVC*		61 ± 8	80 ± 5	81 ± 6	<0.001
Sex^+^	Male	17 (81)	39 (66.1)	38 (71.7)	0.535
	Female	—	—	—	Ref.
Ethnic^+^	Caucasian	14 (66.6)	14 (23.7)	20 (37.7)	0.038
	Black	—	—	—	Ref.
Stage^+^ (Gold) of COPD	II	12 (57.2)	—	—	NA
	I	9 (42.8)	—	—	Ref.

* indicated as mean ± standard deviation, ^+^ indicated as N (%), Ref. considered as the reference level for each categorical variable, NA: not applicable.

### Module identification

**Figure 2:**
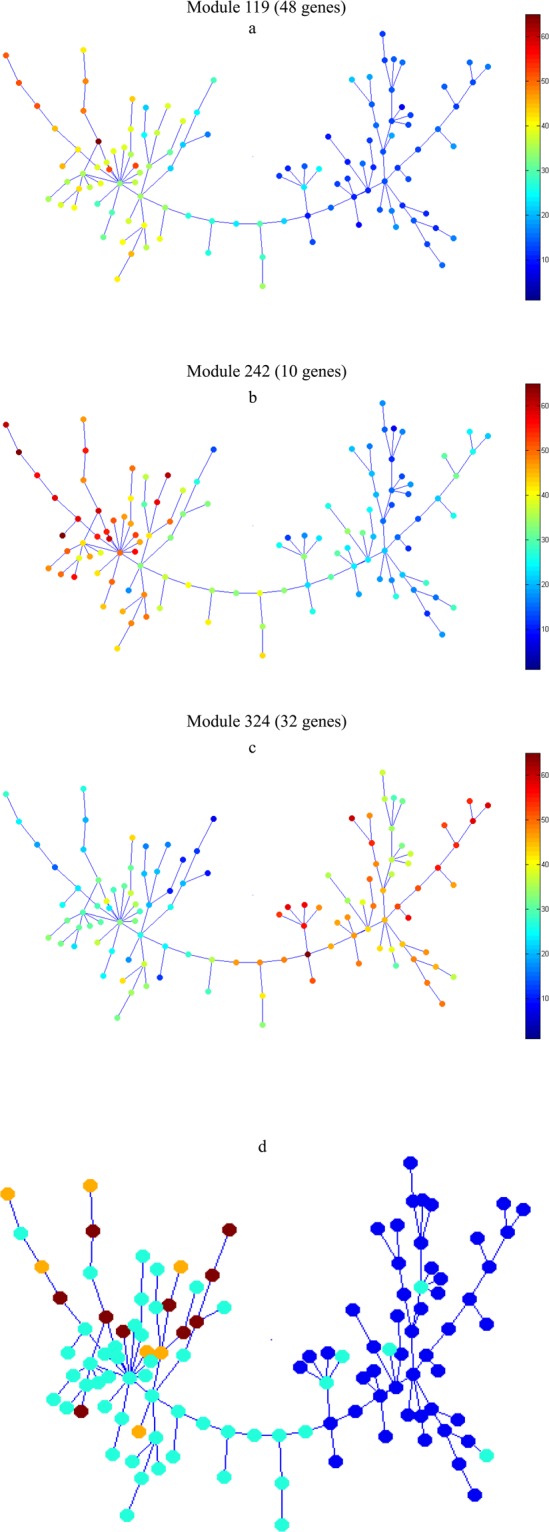
Genes involved in the progression of the COPD based on the minimal- inclusive trees were obtained from SPD algorithm (dark blue = healthy non-smoker, light blue = healthy smoker, light brown = stage I of COPD smoker and dark brown = stage II of COPD smoker).

Normalized gene expression data was used for module identification in the SPD algorithm. In total, 576 modules were identified. Three modules were biologically more related to the progression and phenotype of COPD including, 119, 242 and 324. The minimal spanning trees obtained from the SPD algorithm are shown in Fig. 2. All the genes involved in COPD progression are presented in Table 2 and then included in machine-learning and statistical modeling approaches. From these three selected modules, gene expression within two of the modules (Fig. 2a,b), associated with COPD-progression, was increased in SAE cells. In contrast, gene expression within the third module (Fig. 2c), associated with COPD-progression, was decreased in SAE cells. In Fig. 2d, classification of samples was shown based on the disease stage (dark blue = healthy non-smoker, light blue = healthy smoker, light brown = COPD stage I and dark brown = COPD stage II).

**Table 2:** List of the genes involved in the progression of COPD by sample progression discovery (SPD) algorithm.

Related Modules with progression of COPD	Number of involved Genes	Genes Symbol
Module 119	48	MUCL1, LOC652993, LINC00639, LINC00942, TXNRD1, CYP1B1, ME1, GAD1, CBR3, CYP1A1, NRG1, CYP4F3, AKR1B10, HTR2B, NR0B1, GRM1, ABCC3, CDRT1, AKR1C3, CBR1, TRIM9, SPP1, ADH7, FTH1P5, FTL, ADD3-AS1, AKR1C1, SLC7A11, CACNA2D3, LHX6, CABYR, HS3ST3A1, PLEKHA8P1, BACH2, SFRP2, RPSA, CLIP4, ST3GAL4-AS1, SAMD5, AHRR, ANKDD1A, LINC00589, TMCC3, RNF175, RIMKLA, LOC100652994, GPX2, LOC344887
Module 242	10	LINC00930, UCHL1, REEP1, EGF, CLEC11A, TMEM74B, DNHD1, C4orf48, C6orf164, JAKMIP3
Module 324	32	ZSCAN4, LOC338667, PRKAR2B, PLAG1, ZNF211, SCGB1A1, TLR5, KANK1, PPP4R4, THSD7A, CYB5A, GMNN, GPRC5A, PIEZO2, GFOD1, ZNF419, THSD4, CCDC37, PAPLN, GLI3, PRKAG2-AS1, PRDM11, LOC285812, SCGB3A1, USP27X, KCNA1, LOC100507560, PRDM16, SLITRK6, CYP4Z1, GPR115, RASSF10

### Gene selection and prediction

Based on the machine-learning and statistical penalized algorithms, and after adjustment of the effect of pack per year of smoking, elastic-net logistic regression had the highest AUC (82%), sensitivity (85%), specificity (51%) and lowest misclassification error rate (25%). In reverse, decision trees method has lowest AUC (57%), sensitivity (69%), specificity (43%) and highest misclassification error rate (39%) than other algorithms. Based on the elastic-net logistic regression, the most important selected genes included, THSD4, PPP4R4, JAKMIP3, LINC00930, DNHD1, TMCC3, CCDC37, PRDM11, GLI3, ABCC3, ADH7, SAMD5, RASSF10, USP27X, GAD1, CYP1A1, NR0B1, CYP1B1, PLAG1, PIEZO2, SCGB1A1, LOC100507560.

**Figure 3:**
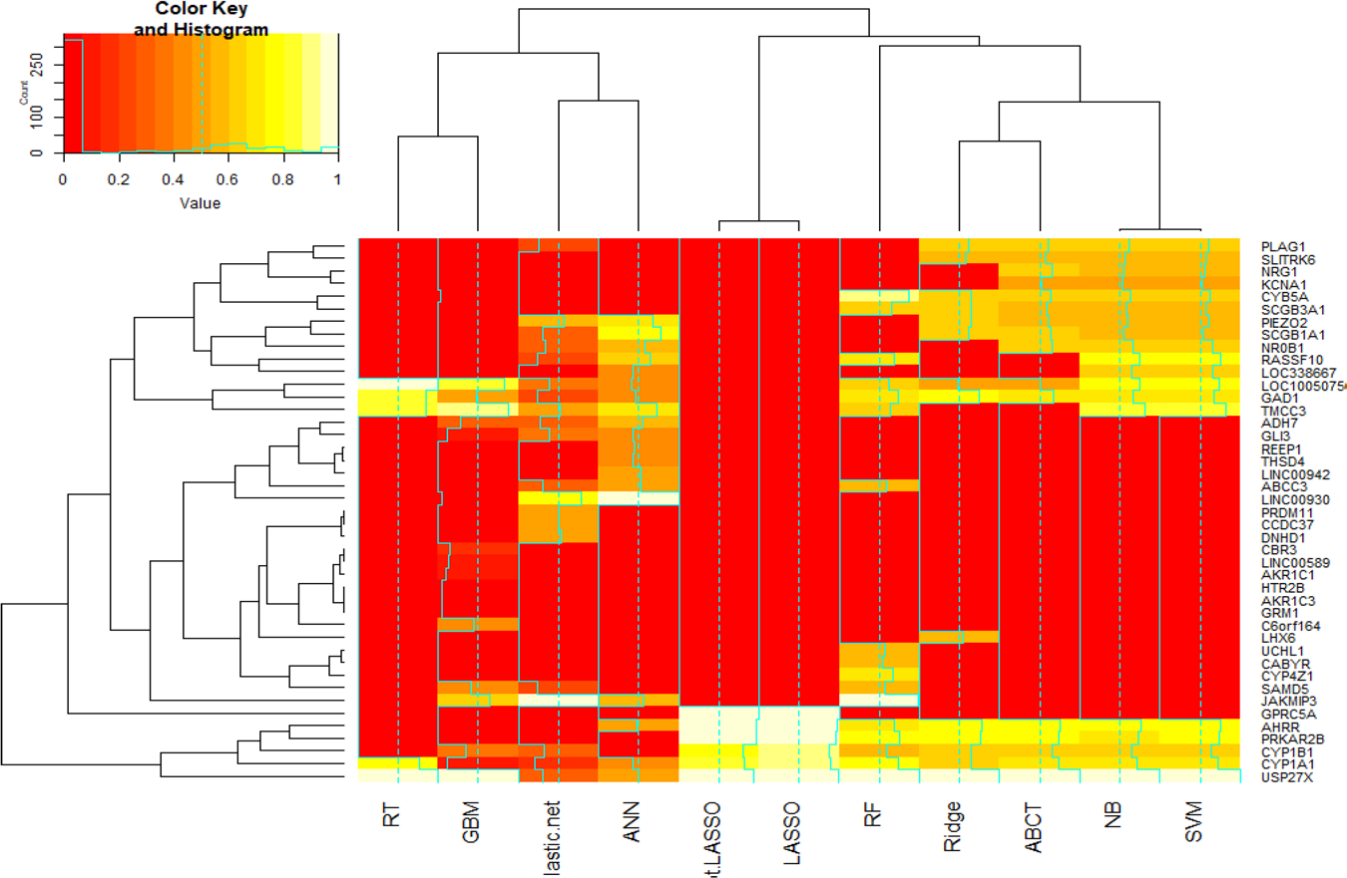
Interactive cluster heatmap displaying importance index of the forty-four candidate genes (as columns) in each of the machine learning and statistical methods (as rows), rows and columns of the heatmap have been reordered according to a hierarchical clustering, represented by the dendrogram, colors represent importance index of the genes (red to yellow: lower to higher of importance value).

**Figure 4:**
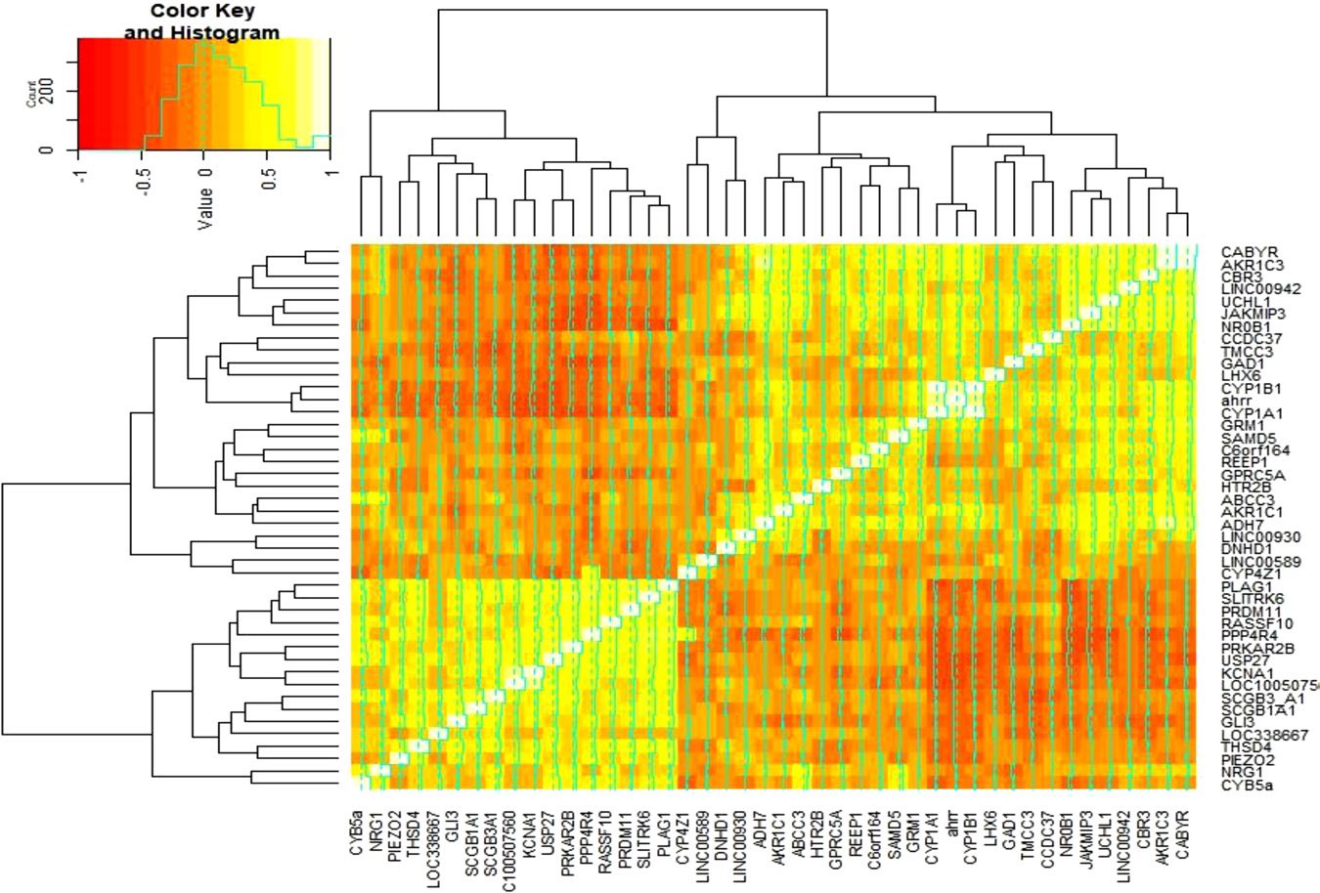
Spearman’s rank correlation, co-expression, matrix between the selected genes: heatmap for hierarchical clustering the forty-four candidate genes based on their pattern of gene expression.

**Figure 5:**
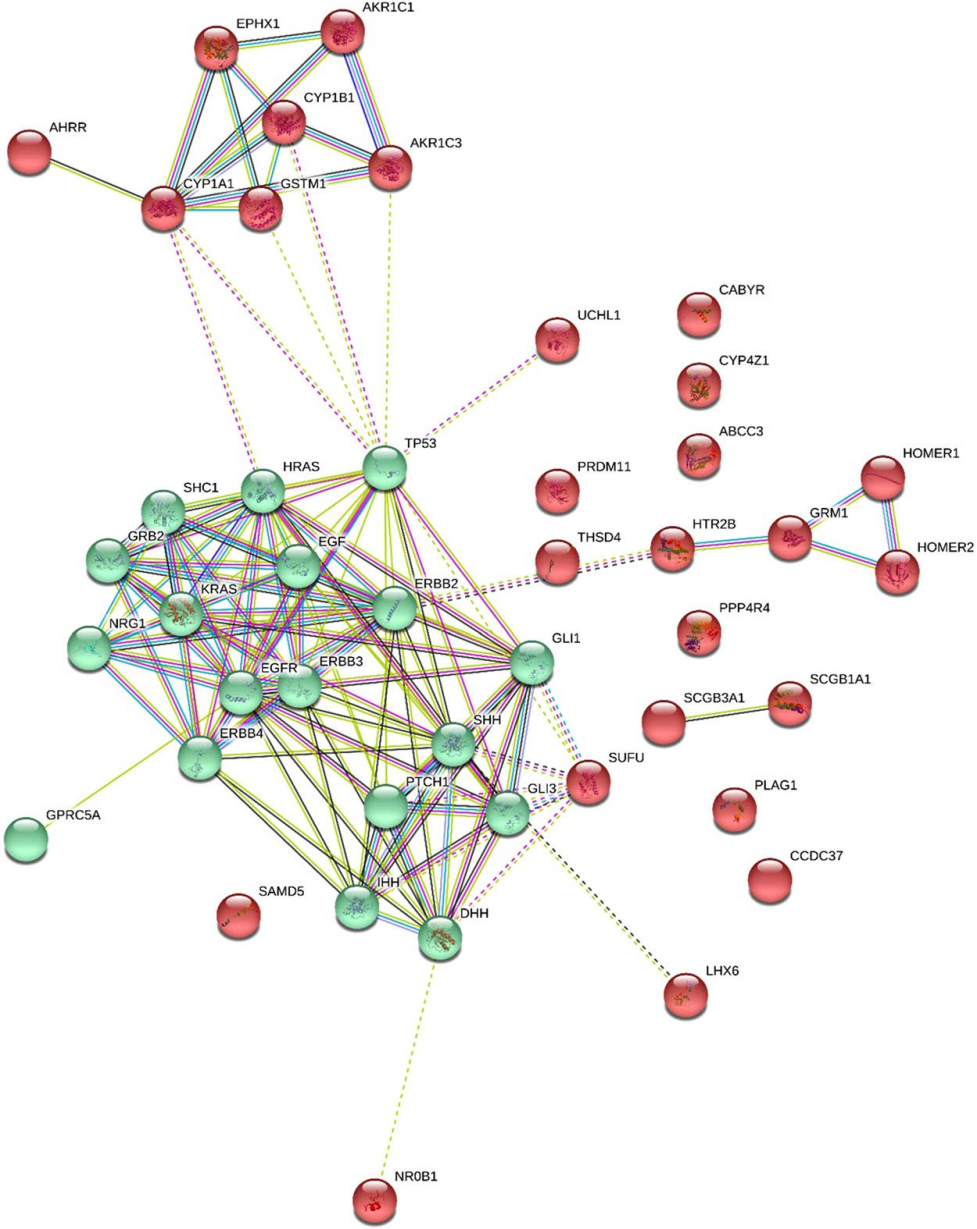
STRING protein–protein interaction networks for the forty-four candidate genes.

Consequently, 44 candidate genes identified here are associated with either the occurrence or progression of COPD, or lung function (Table 3). According to the results of each computational method, 44 were selected and the computational methods were hierarchically clustered, simultaneously (Figs 3 and 4). Of these 44 genes, 27 have been previously reported in the literature to be associated with COPD, lung function (FVC, FEV_1_ or the FEV_1_/FVC ratio) or other lung diseases. These 27 genes also include the genes of THSD4, PPP4R4, SCGB1A1, and NRG1, already detected in GWA studies to determine single nucleotide polymorphisms (SNPs) specifically for COPD (Table 4). Furthermore, in our study, SNPs within 4 additional genes have been detected in GWAS studies carried out previously in lung-related studies including: PRDM11 and AHRR FVC, smoking^15,16^, CYP1A1 childhood bronchitis^17^ and CYP1B1 lung cancer^18^. In this study, we have identified 17 genes which have not previously been detected in COPD studies, these include: LINC00942, REEP1, C6orf164, LINC00589, JAKMIP3, LINC00930, DNHD1, TMCC3, ADH7, PRKAR2B, GAD1, LOC338667, CYB5A, PIEZO2, SLITRK6, KCNA1 and LOC100507560 (Table 4). These genes may represent novel biomarkers in the diagnosis and prognosis of COPD. Figure 5 depicts the functional protein-association networks for the 44 selected genes, as shown by STRING.

**Table 3:** Probes and corresponding 44 genes selected by ML algorithms and penalized regression models for association between the genes with occurrence and progression of COPD. The effect of smoking (pack per year) was adjusted in all of the methods.

	Gene Symbol	Probe ID	Number of Methods	LASSO	Adapt. LASSO	Elastic net	Ridge	SVM	GBM	NB	RF	ANN	RT	ABCT
1.	PPP4R4	233002_at	3	80%	78%	96%	—	—	—	—	—	—	—	—
2.	THSD4	222835_at	2	—	—	90%	—	—	—	—	—	43%	—	—
3.	NRG1	206343_s_at	3	—	—	—	—	55%	—	55%	—	—	—	65%
4.	SCGB1A1	205725_at	6	—	—	30%	61%	54%	—	54%	—	78%	—	64%
5.	AHRR	229354_at	8	98%	96%	—	77%	76%	—	76%	68%	48%	—	76%
6.	CYP1A1	205749_at	11	90%	82%	20%	65%	73%	11%	72%	74%	43%	77%	73%
7.	CYP1B1	202437_s_at	9	88%	80%	32%	65%	64%	35%	64%	58%	—	—	65%
8.	PRDM11	229687_s_at	1	—	—	50%	—	—	—	—	—	—	—	—
9.	CBR3	205379_at	1	—	—	—	—	—	14%	—	—	—	—	—
10.	AKR1C1	217626_at	1	—	—	—	—	—	10%	—	—	—	—	—
11.	AKR1C3	209160_at	1	—	—	—	—	—	5%	—	—	—	—	—
12.	GRM1	207299_s_at	1	—	—	—	—	—	4%	—	—	—	—	—
13.	CYP4Z1	237395_at	1	—	—	—	—	—	—	—	67%	—	—	—
14.	UCHL1	201387_s_at	1	—	—	—	—	—	—	—	57%	—	—	—
15.	CABYR	219928_s_at	1	—	—	—	—	—	—	—	54%	—	—	—
16.	GPRC5A	203108_at	2	100%	100%	—	—	—	—	—	—	—	—	—
17.	CCDC37	243758_at	1	—	—	50%	—	—	—	—	—	—	—	—
18.	GLI3	227376_at	3	—	—	38%	—	—	12%	—	—	43%	—	—
19.	ABCC3	208161_s_at	3	—	—	30%	—	—	—	—	58%	52%	—	—
20.	SAMD5	228653_at	3	—	—	24%	—	—	41%	—	57%	—	—	—
21.	RASSF10	238755_at	5	—	—	23%	—	75%	—	75%	68%	64%	—	—
22.	USP27X	230620_at	11	99%	94%	31%	100%	100%	100%	100%	100%	49%	100%	100%
23.	HTR2B	206638_at	1	—	—	—	—	—	5%	—	—	—	—	—
24.	NR0B1	206645_s_at	5	—	—	33%	—	66%	—	66%	—	58%	—	66%
25.	PLAG1	205372_at	5	—	—	26%	61%	61%	—	61%	—	—	—	61%
26.	SCGB3A1	230378_at	5	—	—	—	65%	58%	—	58%	65%	—	—	58%
27.	LHX6	219884_at	1	—	—	—	55%	—	—	—	—	—	—	—
28.	LINC00942	1558308_at	1	—	—	—	—	—	—	—	—	52%	—	—
29.	REEP1	204364_s_at	1	—	—	—	—	—	—	—	—	45%	—	—
30.	C6orf164	230506_at	1	—	—	—	—	—	44%	—	—	—	—	—
31.	LINC00589	232718_at	1	—	—	—	—	—	13%	—	—	—	—	—
32.	JAKMIP3	233076_at	4	—	—	100%	—	—	64%	—	98%	56%	—	—
33.	LINC00930	1556768_at	3	—	—	78%	—	—	4%	—	—	100%	—	—
34.	DNHD1	229631_at	1	—	—	53%	—	—	—	—	—	—	—	—
35.	TMCC3	235146_at	7	—	—	52%	—	82%	87%	82%	64%	73%	84%	—
36.	ADH7	210505_at	3	—	—	27%	—	—	27%	—	—	54%	—	—
37.	PRKAR2B	203680_at	7	96%	96%	—	76%	74%	—	73%	76%	—	—	74%
38.	GAD1	205278_at	9	—	—	23%	74%	67%	48%	67%	73%	46%	84%	67%
39.	LOC338667	1564786_at	3	—	—	—	—	65%	—	65%	—	43%	—	—
40.	CYB5A	217021_at	6	—	—	—	65%	63%	3%	63%	87%	—	—	64%
41.	PIEZO2	219602_s_at	6	—	—	56%	65%	60%	—	60%	—	68%	—	60%
42.	SLITRK6	235976_at	4	—	—	—	58%	57%	—	57%	—	—	—	57%
43.	KCNA1	230849_at	3	—	—	—	—	52%	—	53%	—	—	—	53%
44.	LOC100507560	231379_at	9	—	—	38%	48%	74%	82%	74%	62%	41%	100%	50%
	AUC% Sensitivity (SD) Specificity (SD) Misclassification Error Rate (SD)			79%	74%	82%	76.6%	61.6%	76%	77%	80%	70%	57%	74.7%
				0.83 (0.14)	0.81 (0.16)	0.85 (0.13)	1	0.92 (0.10)	0.98 (0.04)	0.84 (0.12)	0.95 (0.08)	0.68 (0.17)	0.69 (0.20)	0.81 (0.14)
				0.5 (0.30)	0.37 (0.10)	0.51 (0.29)	0	0.15 (0.13)	0.02 (0.07)	0.49 (0.26)	0.07 (0.15)	0.66 (0.24)	0.43 (0.24)	0.39 (0.14)
				0.27 (0.14)	0.31 (0.15)	0.25 (0.10)	0.30 (0.03)	0.31 (0.06)	0.30 (0.05)	0.26 (0.09)	0.31 (0.09)	0.32 (0.13)	0.39 (0.12)	0.31 (0.11)

Important index (value) for each gene in any method was reported. The third column indicated number of studies that it confirmed the association of each gene with progression of the COPD. Third column indicated sum of number of methods that it confirmed each gene (Range score: 0 to 11).

**Table 4:** Confirmation of the association of selected genes with COPD/or lung function by literature reviewing in PubMed databank with ((“COPD” OR “Lung Function”) AND “name of each selected gene”).

	Gene Symbol	Probe ID	Number of studies	References (PMIDs)
1.	PPP4R4	233002_at	1	28170284
2.	THSD4	222835_at	6	27564456, 24286382, 23932459, 22461431, 21965014, 20010834
3.	NRG1	206343_s_at	15	28950338, 28901268, 28604730, 28396363, 28391773, 27626312, 26837769, 26200269, 25870798, 25531467, 25501131, 25384085, 24469108, 23390248, 22665269
4.	SCGB1A1	205725_at	4	27081700, 26937342, 26159408, 23144326
5.	AHRR	229354_at	9	28854564, 29262847, 28100713, 28056099, 27924164, 27632354, 26667048, 22232023, 18172554
6.	CYP1A1	205749_at	108	29212267, 29076184, 28827732, 28283091, and etc.
7.	CYP1B1	202437_s_at	38	29110844, 28858732, and etc.
8.	PRDM11	229687_s_at	1	28938616
9.	CBR3	205379_at	1	26916823
10.	AKR1C1	217626_at	8	29344298, 28210161, 26338969, 24976539, 23534707, 23474755, 17266043, 16915569
11.	AKR1C3	209160_at	7	23534707, 28704416, 27629782, 25603868, 23665002, 23519145, 15284179
12.	HTR2B	206638_at	1	27301951
13.	GRM1	207299_s_at	1	23303475
14.	CYP4Z1	237395_at	1	19473719
15.	UCHL1	201387_s_at	5	28688920, 25615526, 23534707, 21143527, 17108109
16.	CABYR	219928_s_at	5	26938915, 26843620, 24362251, 17317841, 21274509
17.	GPRC5A	203108_at	10	29382653, 28849235, 28088789, 26447616, 25621293, 25311788, 23154545, 22239913, 20686609, 20563252
18.	CCDC37	243758_at	2	26200272, 22011669
19.	GLI3	227376_at	3	27146893, 23736020, 23667589
20.	ABCC3	208161_s_at	4	24176985, 23369236, 22699933, 19107936
21.	SAMD5	228653_at	1	25411851
22.	RASSF10	238755_at	1	24433832
23.	USP27X	230620_at	1	27013495
24.	NR0B1	206645_s_at	1	28965760
25.	PLAG1	205372_at	2	29305497, 29249655
26.	SCGB3A1	230378_at	5	26937342, 21636547, 20849603, 20660313, 19334046
27.	LHX6	219884_at	4	28900494, 28396596, 27610375, 24157876
28.	LINC00942	1558308_at	0	—
29.	REEP1	204364_s_at	0	—
30.	C6orf164	230506_at	0	—
31.	LINC00589	232718_at	0	—
32.	JAKMIP3	233076_at	0	—
33.	LINC00930	1556768_at	0	—
34.	DNHD1	229631_at	0	—
35.	TMCC3	235146_at	0	—
36.	ADH7	210505_at	0	—
37.	PRKAR2B	203680_at	0	—
38.	GAD1	205278_at	0	—
39.	LOC338667	1564786_at	0	—
40.	CYB5A	217021_at	0	—
41.	PIEZO2	219602_s_at	0	—
42.	SLITRK6	235976_at	0	—
43.	KCNA1	230849_at	0	—
44.	LOC100507560	231379_at	0	—

### Investigation of the differential expression of genes in healthy non-smokers (HNS; control subjects), healthy smokers, COPD patients, and COPD Stage I and II patients

In this study, we also investigated the differential expression of our 44 candidate genes in healthy non-smokers (HNS; control subjects; n = 53), healthy smokers (HS; n = 59), COPD patients (n = 21), and COPD stage I (COPD I; n = 9) and II (COPD II; n = 12) patients, respectively. We investigated the differential gene expression between HNS and HS and found significant differences in expression in 39/44 (88.6%) of all genes. In addition, 16/17 (94.1%) of the genes, not previously detected associating with COPD or lung function, were differentially expressed (Table 5; column HS v HNS). We then investigated the differential expression of these 44 genes in HS and COPD patients. Here, 24/44 (54.5%) of all genes studies were significantly regulated. Furthermore, 10/17 previously undetected genes in COPD/lung function were differentially regulated (Table 5; column COPD v HS). Finally, we investigated the regulation of these 44 genes in COPD Stage I and II patients compared with HS (Table 5; columns stage I v HS and stage II v HS). Here, we observed that 5/44 (11.4%; COPD stage I) and 16/44 (36.3%; COPD stage II) were differentially regulated. Among the previously undetected genes in COPD/lung function, 10/17 (58.8%) and 6/17 (35.3%) were significantly different in COPD stage I and II, respectively, compared with HS. A number of genes were significantly different in all four analyses (HS v HNS; HS v COPD; HS v COPD I; HS v COPD II), including: USP27X, AHRR, CYP1A1 and CYP1B1. Interestingly, of these genes, not previously identified to associate with COPD/lung function, PRKAR2B and GAD1 were significantly different in all four analyses. Therefore, this study reveals for the first time the potential role of PRKAR2B and GAD1 in COPD and smoking-related dysfunction in lung.

**Table 5:** Relative expression of 44 candidate genes in healthy controls (smokers and non-smokers) and COPD smoker patients (stage I and stage II).

	Gene Symbol	Healthy Non-Smoker (N = 53)	Healthy Smoker (N = 59)	COPD smoker (N = 21)	COPD stage I smoker (N = 9)	COPD stage II smoker (N = 12)	Fold Regulation, adjusted p-value (HS vs. HNS)	Fold Regulation, adjusted p-value (COPD vs. HS)	Fold Regulation, adjusted p-value (stage I vs. HS)	Fold Regulation, adjusted p-value (stage II vs. HS)
1.	PPP4R4	6.56 ± 0.75	6.01 ± 0.88	5.56 ± 0.69	5.88 ± 0.44	5.32 ± 0.77	−1.091, 0.004	−1.081, 0.026	−1.022, 0.99	−1.130, 0.036
2.	THSD4	7.59 ± 0.50	7.34 ± 0.54	7.11 ± 0.51	7.47 ± 0.31	6.84 ± 0.46	−1.034, 0.052	−1.032, 0.113	1.018, 0.99	−1.073, 0.013
3.	NRG1	3.79 ± 0.25	4.05 ± 0.47	3.80 ± 0.38	3.94 ± 0.48	3.69 ± 0.25	1.069, 0.002	−1.066, 0.005	−1.028, 0.99	−1.097, 0.021
4.	SCGB1A1	14.48 ± 0.15	14.36 ± 0.20	14.15 ± 0.32	14.18 ± 0.24	14.11 ± 0.37	−1.008, 0.015	−1.015, 0.002	−1.013, 0.103	−1.018, 0.002
5.	AHRR	3.86 ± 0.16	4.58 ± 0.65	5.23 ± 0.76	5.42 ± 0.81	5.08 ± 0.71	1.186, 0.001	1.142, 0.001	1.183, 0.001	1.109, 0.025
6.	CYP1A1	4.61 ± 0.24	6.12 ± 1.84	7.72 ± 1.80	8.04 ± 1.52	7.48 ± 2.02	1.327, 0.001	1.261, 0.001	1.314, 0.003	1.222, 0.018
7.	CYP1B1	3.64 ± 0.51	7.74 ± 2.04	9.50 ± 1.16	9.92 ± 0.59	9.19 ± 1.40	2.126, 0.001	1.227, 0.002	1.282, 0.003	1.187, 0.045
8.	PRDM11	5.67 ± 0.36	5.35 ± 0.32	5.28 ± 0.29	5.34 ± 0.22	5.23 ± 0.33	−1.059, 0.001	−1.013, 0.363	−1.002, 0.99	−1.023, 0.99
9.	CBR3	7.23 ± 0.43	8.15 ± 0.68	8.35 ± 0.64	8.47 ± 0.50	8.26 ± 0.73	1.127, 0.001	1.025, 0.287	1.039, 0.819	1.013, 0.99
10.	AKR1C1	6.82 ± 0.68	8.56 ± 1.11	8.32 ± 1.20	8.44 ± 1.23	8.22 ± 1.21	1.255, 0.001	−1.029, 0.542	−1.014, 0.99	−1.041, 0.99
11.	AKR1C3	10.30 ± 0.43	11.85 ± 0.72	11.96 ± 0.66	12.20 ± 0.47	11.78 ± 0.74	1.150, 0.001	1.009, 0.748	1.029, 0.783	−1.006, 0.99
12.	HTR2B	3.92 ± 0.21	4.21 ± 0.30	4.25 ± 0.31	4.19 ± 0.40	4.29 ± 0.23	1.074, 0.001	1.010, 0.618	−1.005, 0.99	1.019, 0.99
13.	GRM1	3.74 ± 0.11	4.04 ± 0.34	4.16 ± 0.55	4.21 ± 0.36	4.13 ± 0.66	1.080, 0.001	1.030, 0.571	1.042, 0.99	1.022, 0.99
14.	CYP4Z1	6.83 ± 0.57	6.24 ± 0.57	5.92 ± 0.38	5.89 ± 0.45	5.94 ± 0.33	−1.094, 0.001	−1.054, 0.02	−1.059, 0.445	−1.050, 0.404
15.	UCHL1	5.30 ± 0.56	8.79 ± 1.50	9.27 ± 1.81	9.24 ± 1.69	9.28 ± 1.97	1.658, 0.001	1.055, 0.183	1.051, 0.99	1.055, 0.97
16.	CABYR	4.86 ± 0.28	6.92 ± 1.23	7.46 ± 1.37	7.98 ± 1.13	7.09 ± 1.46	1.424, 0.001	1.078, 0.162	1.153, 0.003	1.024, 0.99
17.	GPRC5A	7.58 ± 0.63	7.50 ± 0.43	8.01 ± 0.61	7.89 ± 0.71	8.10 ± 0.54	−1.010, 0.99	1.068, 0.001	1.052, 0.262	1.080, 0.005
18.	CCDC37	9.44 ± 0.54	9.35 ± 0.53	9.26 ± 0.63	9.21 ± 0.80	9.29 ± 0.50	−1.009, 0.99	−1.010, 0.381	−1.015, 0.99	−1.006, 0.99
19.	GLI3	7.59 ± 0.38	6.74 ± 0.57	6.62 ± 0.38	6.69 ± 0.43	6.56 ± 0.34	−1.126, 0.001	−1.018, 0.292	−1.007, 0.99	−1.027, 0.99
20.	ABCC3	6.95 ± 0.44	7.88 ± 0.61	7.62 ± 0.78	7.68 ± 0.76	7.57 ± 0.82	1.134, 0.001	−1.034, 0.226	−1.026, 0.99	−1.041, 0.733
21.	SAMD5	3.74 ± 0.14	3.98 ± 0.28	3.93 ± 0.49	4.0 ± 0.28	3.87 ± 0.62	1.064, 0.001	−1.013, 0.196	1.005, 0.99	−1.028, 0.99
22.	RASSF10	7.67 ± 0.49	7.06 ± 0.59	6.62 ± 0.55	6.80 ± 0.31	6.47 ± 0.66	−1.086, 0.001	−1.066, 0.001	−1.038, 0.99	−1.091, 0.006
23.	USP27X	7.43 ± 0.28	7.13 ± 0.40	6.65 ± 0.40	6.70 ± 0.27	6.60 ± 0.48	−1.042, 0.001	−1.072, 0.001	−1.064, 0.007	−1.080, 0.001
24.	NR0B1	3.93 ± 0.24	4.39 ± 0.77	4.76 ± 0.77	4.87 ± 0.89	4.67 ± 0.69	1.117, 0.001	1.084, 0.025	1.109, 0.152	1.064, 0.960
25.	PLAG1	5.79 ± 0.56	5.25 ± 0.55	4.81 ± 0.54	4.95 ± 0.54	4.70 ± 0.54	−1.103, 0.001	−1.091, 0.002	−1.060, 0.743	−1.117, 0.025
26.	SCGB3A1	14.21 ± 0.41	13.92 ± 0.60	13.16 ± 0.83	13.53 ± 0.91	12.87 ± 0.65	−1.021, 0.061	−1.058, 0.001	−1.029, 0.412	−1.081, 0.001
27.	LHX6	5.36 ± 0.34	5.80 ± 0.44	6.12 ± 0.51	6.11 ± 0.51	6.12 ± 0.53	1.082, 0.001	1.055, 0.029	1.053, 0.246	1.055, 0.147
28.	LINC00942	3.58 ± 0.17	3.87 ± 0.71	4.11 ± 0.99	4.24 ± 1.23	4.01 ± 0.79	1.081, 0.035	1.062, 0.258	1.096, 0.251	1.036, 0.99
29.	REEP1	9.34 ± 0.63	9.82 ± 0.56	9.61 ± 0.77	9.38 ± 0.93	9.78 ± 0.62	1.051, 0.001	−1.022, 0.329	−1.047, 0.408	−1.004, 0.99
30.	C6orf164	5.49 ± 0.50	6.09 ± 0.55	6.21 ± 0.61	6.17 ± 0.43	6.23 ± 0.73	1.109, 0.001	1.020, 0.315	1.013, 0.99	1.023, 0.99
31.	LINC00589	5.15 ± 0.26	5.38 ± 0.31	5.40 ± 0.31	5.33 ± 0.17	5.44 ± 0.38	1.044, 0.001	1.004, 0.706	−1.009, 0.99	1.011, 0.99
32.	JAKMIP3	4.51 ± 0.21	5.27 ± 0.61	5.68 ± 0.85	5.71 ± 0.98	5.65 ± 0.79	1.168, 0.001	1.078, 0.048	1.083, 0.099	1.072, 0.160
33.	LINC00930	6.01 ± 0.43	6.91 ± 0.57	6.54 ± 0.67	6.54 ± 0.82	6.53 ± 0.58	1.150, 0.001	−1.057, 0.059	−1.056, 0.503	−1.058, 0.186
34.	DNHD1	6.64 ± 0.48	7.14 ± 0.53	7.14 ± 0.74	7.14 ± 0.92	7.13 ± 0.61	1.075, 0.001	1.000, 0.912	1, 0.99	−1.001, 0.99
35.	TMCC3	3.82 ± 0.38	4.09 ± 0.48	4.45 ± 0.43	4.49 ± 0.47	4.42 ± 0.42	1.071, 0.007	1.088, 0.001	1.098, 0.088	1.081, 0.149
36.	ADH7	8.01 ± 0.93	10.81 ± 0.69	10.70 ± 0.62	10.81 ± 0.56	10.61 ± 0.68	1.350, 0.001	−1.010, 0.287	1, 0.99	−1.019, 0.99
37.	PRKAR2B	7.35 ± 0.58	6.45 ± 0.72	5.86 ± 0.59	5.89 ± 0.66	5.83 ± 0.56	−1.139, 0.001	−1.101, 0.001	−1.095, 0.096	−1.106, 0.015
38.	GAD1	4.91 ± 0.61	6.31 ± 1.0	7.25 ± 0.86	7.07 ± 1.10	7.39 ± 0.66	1.285, 0.001	1.149, 0.001	1.120, 0.076	1.171, 0.001
39.	LOC338667	5.39 ± 0.32	5.19 ± 0.23	5.04 ± 0.25	5.03 ± 0.17	5.04 ± 0.30	−1.038, 0.002	−1.030, 0.014	−1.032, 0.647	−1.030, 0.675
40.	CYB5A	4.72 ± 0.19	4.71 ± 0.18	4.53 ± 0.24	4.62 ± 0.23	4.45 ± 0.23	−1.003, 0.99	−1.040, 0.002	−1.019, 0.99	−1.058, 0.010
41.	PIEZO2	7.24 ± 0.85	6.48 ± 0.82	5.89 ± 0.63	5.77 ± 0.72	5.96 ± 0.58	−1.117, 0.001	−1.100, 0.004	−1.123, 0.090	−1.087, 0.225
42.	SLITRK6	7.97 ± 0.61	6.86 ± 0.84	6.24 ± 0.78	6.31 ± 1.04	6.18 ± 0.55	−1.162, 0.001	−1.099, 0.004	−1.087, 0.322	−1.110, 0.041
43.	KCNA1	6.87 ± 1.03	5.79 ± 0.94	5.19 ± 0.77	5.49 ± 0.89	4.97 ± 0.51	−1.186, 0.001	−1.116, 0.011	−1.054, 0.99	−1.165, 0.043
44.	LOC100507560	5.85 ± 0.69	5.42 ± 0.61	4.92 ± 0.48	4.92 ± 0.55	4.91 ± 0.44	−1.079, 0.002	−1.102, 0.001	−1.102, 0.155	−1.104, 0.080

The Adj. P is based on the marginally adjusted 𝑝 values by the Benjamini-Hochberg-FDR correction at α = 0.05; Median ± Interquartile range.

### Investigation of the gender effect on differential gene expression in HNS, HS and COPD (Stage I and II) patients

Here we examined the effects of gender on the expression of our 44 candidate genes. We demonstrated that the expression of 40/44 (90.9%) of these genes is significantly different in HS men compared to HNS men (Table 6; HS v HNS). In addition, 15/17 (88.2%) of the novel genes previously undetected in COPD/lung function had significantly different expression levels (Table 6; HS v HNS) in men. Investigation of the expression levels of the 44 candidate genes in men with COPD versus HS revealed that 21/44 (47.7%) of genes were significantly different (Table 6; COPD v HS) and 10/17 (58.8%) of previously undetected genes in COPD/lung function were also significantly different. When HS were compared to COPD Stage I and II patients, respectively, 4/44 (Stage I; 9.0%) and 7/44 (Stage II; 15.9%) of the total candidate genes were significantly different in male HS compared to HNS. Of the 17 novel genes detected in this study, 1/17 (Stage I; 5.9%) and 3/17 (Stage II; 17.6%) were significantly different in males compared to HS (Table 6; Stage I or Stage II v HS). A number of the 44 candidate genes were significantly different in males across all four analyses, these included USP27X, AHRR, and the novel gene, JAKMIP3.

**Table 6:** Comparison of relative expression of 44 candidate genes between healthy controls (smokers and non-smokers) and COPD smoker patients (stage I and stage II) in men and women groups separately.

Sex group		Gene Symbol	Healthy Non-Smoker (N = 37)	Healthy Smoker (N = 38)	COPD smoker (N = 17)	COPD stage I smoker (N = 9)	COPD stage II smoker (N = 8)	Fold Regulation, adjusted p-value (HS vs. HNS)	Fold Regulation, adjusted p-value (COPD vs. HS)	Fold Regulation, adjusted p-value (stage I vs. HS)	Fold Regulation, adjusted p-value (stage II vs. HS)
Men	1.	PPP4R4	6.45 ± 0.77	6.10 ± 0.84	5.66 ± 0.65	5.88 ± 0.44	5.41 ± 0.79	−1.057, 0.395	−1.078, 0.061	−1.038, 0.99	−1.128, 0.14
	2.	THSD4	7.61 ± 0.49	7.28 ± 0.49	7.22 ± 0.44	7.47 ± 0.31	6.95 ± 0.41	−1.044, 0.022	−1.008, 0.61	1.026, 0.99	−1.048, 0.40
	3.	NRG1	3.76 ± 0.22	4.04 ± 0.51	3.82 ± 0.39	3.94 ± 0.48	3.69 ± 0.23	1.073, 0.010	−1.058, 0.032	−1.024, 0.99	−1.095, 0.10
	4.	SCGB1A1	14.49 ± 0.13	14.35 ± 0.21	14.17 ± 0.32	14.19 ± 0.25	14.15 ± 0.41	−1.010, 0.030	−1.013, 0.035	−1.011, 0.260	−1.014, 0.138
	5.	AHRR	3.87 ± 0.17	4.67 ± 0.67	5.33 ±± 0.79	5.43 ± 0.81	5.23 ± 0.81	1.206, 0.001	1.141, 0.003	1.162, 0.002	1.120, 0.064
	6.	CYP1A1	4.63 ± 0.24	6.31 ± 1.84	7.73 ± 1.61	8.04 ± 1.52	7.40 ± 1.75	1.364, 0.001	1.225, 0.005	1.274, 0.011	1.172, 0.318
	7.	CYP1B1	3.70 ± 0.56	8.04 ± 2.05	9.68 ± 0.88	9.92 ± 0.60	9.42 ± 1.11	2.174, 0.001	1.204, 0.013	1.234, 0.019	1.172, 0.246
	8.	PRDM11	5.67 ± 0.35	5.33 ± 0.34	5.31 ± 0.30	5.35 ± 0.22	5.27 ± 0.39	−1.062, 0.001	−1.004, 0.827	1.003, 0.99	−1.012, 0.99
	9.	CBR3	7.27 ± 0.44	8.27 ± 0.65	8.44 ± 0.64	8.48 ± 0.50	8.40 ± 0.80	1.137, 0.001	1.021, 0.392	1.026, 0.99	1.017, 0.99
	10.	AKR1C1	6.74 ± 0.62	8.61 ± 1.10	8.55 ± 1.09	8.44 ±± 1.24	8.67 ± 0.97	1.277, 0.001	−1.007, 0.884	−1.019, 0.99	1.007, 0.99
	11.	AKR1C3	10.28 ± 0.41	11.9 ± 50.76	12.06 ± 0.60	12.20 ± 0.47	11.91 ± 0.73	1.162, 0.001	1.013, 0.956	1.021, 0.99	−1.004, 0.99
	12.	HTR2B	3.86 ± 0.17	4.26 ± 0.30	4.24 ± 0.32	4.19 ± 0.40	4.30 ± 0.21	1.104, 0.001	−1.005, 0.884	−1.016, 0.99	1.009, 0.99
	13.	GRM1	3.74 ± 0.12	4.06 ± 0.35	4.19 ± 0.59	4.20 ± 0.37	4.17 ± 0.81	1.083, 0.002	1.032, 0.478	1.036, 0.99	1.028, 0.99
	14.	CYP4Z1	6.81 ± 0.59	6.18 ± 0.54	5.94 ± 0.35	5.90 ± 0.45	5.99 ± 0.21	−1.102, 0.002	−1.040, 0.101	−1.048, 0.88	−1.032, 0.99
	15.	UCHL1	5.30 ± 0.62	8.67 ± 1.37	9.46 ± 1.63	9.25 ± 1.69	9.72 ± 1.63	1.635, 0.001	1.091, 0.061	1.066, 0.99	1.121, 0.13
	16.	CABYR	4.86 ± 0.28	7.14 ± 1.18	7.60 ± 1.39	7.98 ± 1.13	7.20 ± 1.63	1.470, 0.001	1.064, 0.308	1.118, 0.15	1.008, 0.99
	17.	GPRC5A	7.64 ± 0.61	7.57 ± 0.43	8.02 ± 0.62	7.89 ± 0.71	8.17 ± 0.52	−0.990, 0.99	1.059, 0.006	1.043, 0.57	1.080, 0.032
	18.	CCDC37	9.48 ± 0.55	9.29 ± 0.57	9.25 ± 0.65	9.22 ± 0.80	9.28 ± 0.47	−1.021, 0.969	−1.004, 0.61	−1.008, 0.99	−1.001, 0.99
	19.	GLI3	7.55 ± 0.39	6.62 ± 0.55	6.60 ± 0.41	6.70 ± 0.43	6.50 ± 0.39	−1.141, 0.001	−1.003, 0.927	1.013, 0.99	−1.018, 0.99
	20.	ABCC3	6.97 ± 0.46	7.95 ± 0.54	7.72 ± 0.77	7.68 ± 0.76	7.77 ± 0.83	1.140, 0.001	−1.030, 0.412	−1.034, 0.99	−1.023, 0.99
	21.	SAMD5	3.74 ± 0.14	4.02 ± 0.30	3.94 ± 0.54	4.00 ± 0.28	3.87 ± 0.76	1.074, 0.002	−1.020, 0.133	−1.003, 0.99	−1.037, 0.99
	22.	RASSF10	7.70 ± 0.44	7.12 ± 0.57	6.67 ± 0.52	6.81 ± 0.31	6.51 ± 0.67	−1.082, 0.001	−1.067, 0.004	−1.045, 0.552	−1.093, 0.02
	23.	USP27X	7.43 ± 0.29	7.12 ± 0.39	6.68 ± 0.28	6.71 ± 0.27	6.65 ± 0.30	−1.043, 0.001	−1.066, 0.001	−1.061, 0.007	−1.071, 0.002
	24.	NR0B1	3.94 ± 0.24	4.39 ± 0.78	4.84 ± 0.76	4.88 ± 0.89	4.80 ± 0.64	1.114, 0.005	1.103, 0.011	1.112, 0.181	1.094, 0.627
	25.	PLAG1	5.73 ± 0.61	5.22 ± 0.56	4.83 ± 0.57	4.95 ± 0.55	4.69 ± 0.60	−1.098, 0.002	−1.081, 0.018	−1.055, 0.99	−1.113, 0.133
	26.	SCGB3A1	14.25 ± 0.34	13.86 ± 0.64	13.31 ± 0.84	13.54 ± 0.91	13.07 ± 0.72	−1.028, 0.036	−1.041, 0.025	−1.024, 0.93	−1.060, 0.004
	27.	LHX6	5.36 ± 0.33	5.85 ± 0.38	6.10 ± 0.49	6.12 ± 0.52	6.07 ± 0.48	1.091, 0.001	1.043, 0.071	1.046, 0.336	1.038, 0.754
	28.	LINC00942	3.58 ± 0.19	3.88 ± 0.80	4.21 ± 1.05	4.25 ± 1.24	4.17 ± 0.87	1.084, 0.172	1.085, 0.155	1.094,0.551	1.073, 0.99
	29.	REEP1	9.29 ± 0.66	9.81 ± 0.56	9.62 ± 0.78	9.39 ± 0.93	9.88 ±0.54	1.056, 0.005	−1.020, 0.434	−1.045, 0.581	1.007, 0.99
	30.	C6orf164	5.45 ± 0.42	6.14 ± 0.55	6.23 ± 0.52	6.17 ± 0.43	6.29 ± 0.62	1.126, 0.001	1.015, 0.489	1.005, 0.99	1.024, 0.99
	31.	LINC00589	5.09 ± 0.22	5.36 ± 0.34	5.34 ± 0.28	5.34 ± 0.17	5.35 ± 0.39	1.053, 0.001	−1.004, 0.899	−1.004, 0.99	−1.002, 0.99
	32.	JAKMIP3	4.50 ± 0.20	5.28 ± 0.56	5.75 ± 0.85	5.71 ± 0.98	5.79 ± 0.74	1.173, 0.001	1.089, 0.049	1.082, 0.09	1.096, 0.07
	33.	LINC00930	6.01 ± 0.42	6.76 ± 0.53	6.56 ± 0.64	6.55 ± 0.82	6.58 ± 0.41	1.126, 0.001	−1.030, 0.334	−1.033, 0.99	−1.028, 0.99
	34.	DNHD1	6.66 ± 0.50	7.07 ± 0.55	7.16 ± 0.80	7.15 ± 0.93	7.18 ± 0.69	1.061, 0.020	1.013, 0.662	1.011, 0.99	1.015, 0.99
	35.	TMCC3	3.83 ± 0.43	4.15 ± 0.52	4.55 ± 0.42	4.49 ± 0.47	4.61 ± 0.38	1.083, 0.021	1.096, 0.002	1.084, 0.323	1.112, 0.095
	36.	ADH7	7.94 ± 0.88	10.9 ± 10.74	10.78 ± 0.54	10.82 ± 0.56	10.73 ± 0.56	1.375, 0.001	−1.011, 0.166	−1.009, 0.99	−1.017, 0.99
	37.	PRKAR2B	7.26 ± 0.63	6.43 ± 0.71	5.84 ± 0.57	5.89 ± 0.66	5.79 ± 0.50	−1.128, 0.001	−1.101, 0.004	−1.092, 0.161	−1.112, 0.066
	38.	GAD1	4.89 ± 0.60	6.33 ± 1.09	7.23 ± 0.89	7.07 ± 1.10	7.41 ± 0.61	1.295, 0.001	1.142, 0.009	1.117, 0.162	1.171, 0.024
	39.	LOC338667	5.37 ± 0.33	5.17 ± 0.23	4.98 ± 0.21	5.03 ± 0.18	4.92 ± 0.24	−1.040, 0.006	−1.038, 0.012	−1.027, 0.99	−1.050, 0.134
	40.	CYB5A	4.7 ± 0.20	4.72 ± 0.18	4.58 ± 0.20	4.62 ± 0.23	4.53 ± 0.17	1.0, 0.99	−1.031, 0.014	−1.021, 0.99	−1.041, 0.089
	41.	PIEZO2	7.13 ± 0.86	6.34 ± 0.73	5.83 ± 0.63	5.78 ± 0.72	5.89 ± 0.55	−1.125, 0.001	−1.087, 0.02	−1.097, 0.326	−1.077, 0.735
	42.	SLITRK6	7.95 ± 0.65	6.89 ± 0.78	6.34 ± 0.83	6.32 ± 1.04	6.36 ± 0.57	−1.155, 0.001	−1.087, 0.028	−1.090, 0.317	−1.082, 0.379
	43.	KCNA1	6.80 ± 1.00	5.84 ± 0.90	5.34 ± 0.73	5.50 ± 0.90	5.16 ± 0.45	−1.166, 0.001	−1.094, 0.041	−1.062, 0.99	−1.131, 0.271
	44.	LOC100507560	5.85 ± 0.69	5.39 ± 0.60	4.95 ± 0.52	4.92 ± 0.56	4.98 ± 0.50	−1.084, 0.012	−1.089, 0.004	−1.096, 0.263	−1.083, 0.507
		**Gene Symbol**	**Healthy Non-Smoker (N = 16)**	**Healthy Smoker (N = 21)**	**COPD smoker (N = 4)**	**COPD stage I smoker (N = 0)**	**COPD stage II smoker (N = 4)**	**Fold Regulation**, **adjusted p-value (HS vs**. **HNS)**	**Fold Regulation**, **adjusted p-value (COPD vs**. **HS)**	**Fold Regulation**, **adjusted p-value (stage I vs**. **HS)**	**Fold Regulation**, **adjusted p-value (stage II vs**. **HS)**
Women	1.	PPP4R4	6.87 ±± 0.63	5.84 ± 0.96	5.15 ± 0.81	—	5.15 ± 0.81	−1.175, 0.005	−1.135, 0.034	—	−1.135, 0.034
	2.	THSD4	7.58 ± 0.54	7.44 ± 0.63	6.63 ± 0.56	—	6.63 ± 0.56	−1.018, 0.99	−1.123, 0.047	—	−1.123, 0.047
	3.	NRG1	3.87 ± 0.30	4.07 ± 0.39	3.71 ± 0.34	—	3.71 ± 0.34	1.051, 0.291	−1.099, 0.184	—	−1.099, 0.184
	4.	SCGB1A1	14.48 ± 0.20	14.40 ± 0.16	14.04 ± 0.31	—	14.04 ± 0.31	−1.006, 0.682	−1.025, 0.006	—	−1.025, 0.006
	5.	AHRR	3.84 ± 0.16	4.43 ± 0.61	4.81 ± 0.38	—	4.81 ± 0.38	1.154, 0.002	1.085, 0.542	—	1.085, 0.542
	6.	CYP1A1	4.59 ± 0.26	5.79 ± 1.85	7.66 ± 2.80	—	7.66 ± 2.80	1.260, 0.037	1.324, 0.081	—	1.324, 0.081
	7.	CYP1B1	3.52 ± 0.35	7.21 ± 1.96	8.77 ± 2.04	—	8.77 ± 2.04	2.050, 0.001	1.216, 0.27	—	1.216, 0.27
	8.	PRDM11	5.70 ± 0.39	5.39 ± 0.31	5.15 ± 0.18	—	5.15 ± 0.18	−1.057, 0.029	−1.047, 0.56	—	−1.047, 0.56
	9.	CBR3	7.14 ± 0.39	7.93 ± 0.70	7.98 ± 0.53	—	7.98 ± 0.53	1.111, 0.002	1.006, 0.99	—	1.006, 0.99
Women	10.	AKR1C1	7.00 ± 0.83	8.48 ± 1.17	7.40 ± 1.35	—	7.40 ± 1.35	1.211, 0.002	−1.146, 0.224	—	−1.146, 0.224
	11.	AKR1C3	10.36 ± 0.52	11.68 ± 0.61	11.55 ± 0.83	—	11.55 ± 0.83	1.127, 0.002	−1.011, 0.99	—	−1.011, 0.99
	12.	HTR2B	4.08 ± 0.22	4.13 ± 0.31	4.29 ± 0.31	—	4.29 ± 0.31	1.012, 0.99	1.037, 0.97	—	1.037, 0.97
	13.	GRM1	3.75 ± 0.11	4.04 ± 0.34	4.05 ± 0.32	—	4.05 ± 0.32	1.076, 0.010	1.004, 0.99	—	1.004, 0.99
	14.	CYP4Z1	6.91 ± 0.54	6.36 ± 0.63	5.84 ± 0.52	—	5.84 ± 0.52	−1.085, 0.037	−1.090, 0.321	—	−1.090, 0.321
	15.	UCHL1	5.32 ± 0.41	9.03 ± 1.72	8.49 ± 2.66	—	8.49 ± 2.66	1.698, 0.001	−1.063, 0.99	—	−1.063, 0.99
	16.	CABYR	4.87 ± 0.32	6.55 ± 1.26	6.88 ± 1.26	—	6.88 ± 1.26	1.343, 0.001	1.051, 0.99	—	1.051, 0.99
	17.	GPRC5A	7.42 ± 0.69	7.39 ± 0.43	7.96 ± 0.64	—	7.96 ± 0.64	−1.005, 0.99	1.077, 0.216	—	1.077, 0.216
	18.	CCDC37	9.36 ± 0.54	9.48 ± 0.44	9.34 ± 0.62	—	9.34 ± 0.62	1.013, 0.99	−1.015, 0.99	—	−1.015, 0.99
	19.	GLI3	7.70 ± 0.36	6.99 ± 0.55	6.71 ± 0.22	—	6.71 ± 0.22	−1.101, 0.001	−1.042, 0.723	—	−1.042, 0.723
	20.	ABCC3	6.91 ± 0.43	7.76 ± 0.72	7.20 ± 0.78	—	7.20 ± 0.78	1.122, 0.002	−1.077, 0.345	—	−1.077, 0.345
	21.	SAMD5	3.78 ± 0.17	3.91 ± 0.25	3.87 ± 0.21	—	3.87 ± 0.21	1.037, 0.20	−1.010, 0.99	—	−1.010, 0.99
	22.	RASSF10	7.64 ± 0.62	6.96 ± 0.62	6.41 ± 0.74	—	6.41 ± 0.74	−1.097, 0.011	−1.086, 0.374	—	−1.086, 0.374
	23.	USP27X	7.47 ± 0.30	7.16 ± 0.44	6.51 ± 0.81	—	6.51 ± 0.81	−1.043, 0.158	−1.10, 0.04	—	−1.10, 0.04
	24.	NR0B1	3.94 ± 0.24	4.40 ± 0.77	4.44 ± 0.84	—	4.44 ± 0.84	1.119, 0.065	1.009, 0.99	—	1.009, 0.99
	25.	PLAG1	5.95 ± 0.40	5.32 ± 0.55	4.74 ± 0.49	—	4.74 ± 0.49	−1.120, 0.002	−1.121, 0.115	—	−1.121, 0.115
	26.	SCGB3A1	14.13 ± 0.55	14.05 ± 0.51	12.51 ± 0.26	—	12.51 ± 0.26	−1.006, 0.99	−1.123, 0.001	—	−1.123, 0.001
	27.	LHX6	5.37 ± 0.40	5.73 ± 0.55	6.22 ± 0.68	—	6.22 ± 0.68	1.067, 0.116	1.086, 0.253	—	1.086, 0.253
	28.	LINC00942	3.59 ± 0.14	3.85 ± 0.51	3.73 ± 0.63	—	3.73 ± 0.63	1.074, 0.154	−1.034, 0.99	—	−1.034, 0.99
	29.	REEP1	9.47 ± 0.58	9.85 ± 0.57	9.59 ± 0.81	—	9.59 ± 0.81	1.040, 0.217	−1.028, 0.99	—	−1.028, 0.99
	30.	C6orf164	5.58 ± 0.67	6.01 ± 0.57	6.14 ± 1.02	—	6.14 ± 1.02	1.077, 0.224	1.021, 0.99	—	1.021, 0.99
	31.	LINC00589	5.30 ± 0.29	5.43 ± 0.25	5.64 ± 0.34	—	5.64 ± 0.34	1.025, 0.524	1.037, 0.549	—	1.037, 0.549
	32.	JAKMIP3	4.54 ± 0.23	5.28 ± 0.72	5.39 ± 0.94	—	5.39 ± 0.94	1.164, 0.002	1.020, 0.99	—	1.020, 0.99
	33.	LINC00930	6.04 ± 0.47	7.20 ± 0.56	6.45 ± 0.92	—	6.45 ± 0.92	1.191, 0.001	−1.116, 0.07	—	−1.116, 0.07
	34.	DNHD1	6.61 ± 0.46	7.28 ± 0.47	7.05 ± 0.47	—	7.05 ± 0.47	1.102, 0.001	−1.033, 0.99	—	−1.033, 0.99
	35.	TMCC3	3.82 ± 0.27	4.01 ± 0.40	4.07 ± 0.23	—	4.07 ± 0.23	1.051, 0.283	1.016, 0.99	—	1.016, 0.99
	36.	ADH7	8.22 ± 1.05	10.63 ± 0.57	10.37 ± 0.92	—	10.37 ± 0.92	1.294, 0.001	−1.025, 0.99	—	−1.025, 0.99
	37.	PRKAR2B	7.59 ± 0.37	6.49 ± 0.75	5.92 ± 0.76	—	5.92 ± 0.76	−1.169, 0.001	—1.096, 0.325	—	−1.096, 0.325
	38.	GAD1	4.98 ± 0.66	6.27 ± 0.86	7.35 ± 0.87	—	7.35 ± 0.87	1.260, 0.001	1.171, 0.058	—	1.171, 0.058
	39.	LOC338667	5.45 ± 0.33	5.24 ± 0.25	5.31 ± 0.26	—	5.31 ± 0.26	−1.040, 0.11	1.013, 0.99	—	1.013, 0.99
	40.	CYB5A	4.74 ± 0.19	4.69 ± 0.20	4.31 ± 0.30	—	4.31 ± 0.30	−1.010, 0.99	−1.089, 0.005	—	−1.089, 0.005
	41.	PIEZO2	7.53 ± 0.82	6.78 ±± 0.92	6.14 ± 0.69	—	6.14 ± 0.69	−1.110, 0.054	−1.105, 0.488	—	−1.105, 0.488
	42.	SLITRK6	8.02 ± 0.51	6.82 ± 0.99	5.85 ± 0.34	—	5.85 ± 0.34	−1.176, 0.001	−1.166, 0.069	—	−1.166, 0.069
	43.	KCNA1	7.04 ± 1.15	5.72 ± 1.02	4.63 ± 0.77	—	4.63 ± 0.77	−1.231, 0.002	−1.234, 0.184	—	−1.234, 0.184
	44.	LOC100507560	5.88 ± 0.73	5.48 ± 0.63	4.81 ± 0.31	—	4.81 ± 0.31	−1.073, 0.227	−1.141, 0.171	—	−1.141, 0.171

The Adj. P is based on the marginally adjusted 𝑝 values by the Benjamini-Hochberg-FDR correction at α = 0.05; Median ± Interquartile range.

We then investigated the expression of our 44 candidate genes, including our 17 novel genes, in HNS, HS, COPD, and COPD Stage I and II. Here, we determined that 52.3% of the 44 candidate genes were significantly differentially expressed in HS compared to HNS females (Table 6; HS v HNS). In addition, 47.1% of the 17 novel genes were significantly different in HS females compared to HNS females. A comparison of female COPD patients to HS females revealed that expression of 7/44 (15.9%) of the 44 candidate genes and 2/17 (11.8%) of the 17 novel genes were significantly different in COPD patients compared to HS (Table 6; COPD v HS). Furthermore, we also observed of significant difference in COPD Stage II compared with HS in 6/44 (13.6%) of the 44 candidate genes and 2/17 (11.8%) of the novel genes detected in this study in females (Table 6; Stage II v HS). A number of the 44 candidate genes were significantly different in females across all four analyses, these included CYP1A1 and the novel genes, LINC00930, GAD1 and SLITRK6.

### Investigation of the age effect on differential gene expression in HNS, HS and COPD (Stage I and II) patients

In this study, we also investigated the effect of age (i.e. subject or patient age: < or ≥50 years) on gene expression in HNS, HS, COPD patients, and stage I and II patients. We observed a significant change in the gene expression of 40/44 (90.9%) total candidate genes and 15/17 (88.2%) novel genes in subjects ≤ 50 years (Table 7; <50 years; HS v HNS). Comparison of HS to COPD patients revealed that expression of 20/44 (45.5%) of our candidate genes were significantly different in COPD patients ≤ 50 years. In addition, expression of 8/17 (47.1%) of our novel genes were significantly different in COPD patients compared to HS ≤ 50 years (Table 7; <50 years; COPD v HS). We also investigated differential gene expression in Stage I and II COPD patients ≤ 50 years and determined that 6/44 (13.6%; Stage I) and 9/44 (20.5%; Stage II) candidate genes, respectively, were significantly different in patients ≤ 50 years. In our cohort of 17 novel genes, we determined that 2/17 (11.8%; Stage I) and 3/17 (17.6%; Stage II) among our total 44 candidate genes, respectively, were significantly different in patients ≤ 50 years (Table 7; <50 years; Stage I and Stage II v HS). Furthermore, a certain number of these candidate genes were significantly different in subjects ≤ 50 years, across all four analysis groups, which included USP27X, CYP1A1 and the novel genes of JAKMIP3 and GAD1.

**Table 7:** Comparison of relative expression of 44 candidate genes between healthy controls (smokers and non-smokers) and COPD smoker patients (stage I and stage II) in age groups separately.

Age group		Gene Symbol	Healthy Non-Smoker (N = 45)	Healthy Smoker (N = 50)	COPD smoker (N = 10)	COPD stage I smoker (N = 5)	COPD stage II smoker (N = 5)	Fold Regulation, adjusted p-value (HS vs. HNS)	Fold Regulation, adjusted p-value (COPD vs. HS)	Fold Regulation, adjusted p-value (stage I vs. HS)	Fold Regulation, adjusted p-value (stage II vs. HS)
<50 years old	1.	PPP4R4	6.56 ± 0.77	6.01 ± 0.86	5.23 ± 0.60	5.64 ± 0.43	4.84 ± 0.48	−1.091,0.009	−1.149, 0.005	−1.065, 0.99	−1.242, 0.009
	2.	THSD4	7.65 ± 0.47	7.36 ± 0.56	7.24 ± 0.47	7.47 ± 0.33	7.02 ± 0.51	−1.039, 0.049	−1.017, 0.579	1.015, 0.99	−1.049, 0.91
	3.	NRG1	3.81 ± 0.26	4.05 ± 0.45	3.89 ± 0.50	4.06 ± 0.62	3.72 ± 0.29	1.064, 0.008	−1.041, 0.131	1.002, 0.99	−1.090, 0.336
	4.	SCGB1A1	14.50 ± 0.15	14.36 ± 0.19	14.01 ± 0.41	14.15 ± 0.33	13.88 ± 0.47	−1.010, 0.005	−1.025, 0.009	−1.015, 0.149	−1.035, 0.001
	5.	AHRR	3.87 ± 0.15	4.53 ± 0.63	5.27 ± 0.73	5.66 ± 0.71	4.91 ± 0.59	1.168, 0.001	1.163, 0.004	1.250, 0.001	1.085, 0.618
	6.	CYP1A1	4.61 ± 0.24	5.95 ± 1.79	7.91 ± 2.16	8.22 ± 1.81	7.61 ± 2.67	1.290, 0.001	1.329, 0.008	1.381, 0.007	1.278, 0.046
	7.	CYP1B1	3.64 ± 0.53	7.54 ± 2.04	9.75 ± 1.17	10.23 ± 0.54	9.30 ± 1.53	2.072, 0.001	1.293, 0.003	1.357, 0.006	1.234, 0.17
	8.	PRDM11	5.72 ± 0.36	5.36 ± 0.34	5.20 ± 0.31	5.30 ± 0.18	5.11 ± 0.40	−1.067, 0.001	−1.031, 0.193	−1.011, 0.99	−1.048, 0.82
	9.	CBR3	7.26 ± 0.43	8.10 ± 0.68	8.58 ± 0.59	8.74 ± 0.41	8.44 ± 0.75	1.116, 0.001	1.059, 0.039	1.079, 0.155	1.042, 0.99
	10.	AKR1C1	6.92 ± 0.67	8.54 ± 1.14	8.71 ± 1.18	8.58 ± 1.42	8.85 ± 1.04	1.235, 0.001	1.020, 0.491	1.004, 0.99	1.036, 0.99
	11.	AKR1C3	10.31 ± 0.46	11.82 ± 0.73	12.15 ± 0.67	12.42 ± 0.42	11.88 ± 0.82	1.146, 0.001	1.028, 0.275	1.051, 0.266	1.006, 0.99
	12.	HTR2B	3.94 ± 0.21	4.22 ± 0.32	4.29 ± 0.36	4.31 ± 0.51	4.26 ± 0.18	1.072, 0.001	1.017, 0.421	1.021, 0.99	1.010, 0.99
	13.	GRM1	3.74 ± 0.11	4.01 ± 0.30	4.37 ± 0.73	4.38 ± 0.40	4.36 ± 1.01	1.072, 0.001	1.090, 0.112	1.091, 0.068	1.087, 0.028
	14.	CYP4Z1	6.88 ± 0.58	6.27 ± 0.59	5.90 ± 0.35	5.89 ± 0.45	5.91 ± 0.27	−1.097, 0.001	−1.063, 0.058	−1.065, 0.834	−1.062, 0.903
	15.	UCHL1	5.33 ± 0.59	8.71 ± 1.50	10.11 ± 1.79	10.35 ± 1.36	9.88 ± 2.31	1.636, 0.001	1.161, 0.008	1.188, 0.049	1.134, 0.181
	16.	CABYR	4.86 ± 0.29	6.85 ± 1.20	7.84 ± 1.64	8.52 ± 1.12	7.22 ± 1.99	1.409, 0.001	1.145, 0.037	1.244, 0.003	1.054, 0.99
	17.	GPRC5A	7.58 ± 0.66	7.50 ± 0.45	8.16 ± 0.61	7.98 ± 0.68	8.35 ± 0.55	−1.011, 0.99	1.088, 0.003	1.065, 0.392	1.114, 0.010
	18.	CCDC37	9.47 ± 0.55	9.34 ± 0.55	9.54 ± 0.66	9.40 ± 0.80	9.68 ± 0.55	−1.013, 0.99	1.021, 0.433	1.006, 0.99	1.036, 0.99
	19.	GLI3	7.62 ± 0.39	6.72 ± 0.60	6.51 ± 0.48	6.61 ± 0.49	6.41 ± 0.49	−1.133, 0.001	−1.032, 0.221	−1.017, 0.99	−1.050, 0.99
	20.	ABCC3	6.96 ± 0.43	7.91 ± 0.62	7.98 ± 0.69	8.14 ± 0.66	7.82 ± 0.76	1.136, 0.001	1.009, 0.716	1.029, 0.99	−1.011, 0.99
	21.	SAMD5	3.74 ± 0.15	3.97 ± 0.28	4.13 ± 0.62	4.13 ± 0.24	4.13 ± 0.89	1.060, 0.001	1.040, 0.455	1.041, 0.99	1.041, 0.614
	22.	RASSF10	7.71 ± 0.50	7.08 ± 0.57	6.41 ± 0.60	6.68 ± 0.36	6.15 ± 0.72	−1.089, 0.001	−1.105, 0.002	−1.060, 0.62	−1.152, 0.002
	23.	USP27X	7.46 ± 0.28	7.16 ± 0.40	6.52 ± 0.50	6.74 ± 0.34	6.31 ± 0.58	−1.041, 0.002	−1.098, 0.001	−1.063, 0.075	−1.136, 0.001
	24.	NR0B1	3.95 ± 0.25	4.36 ± 0.78	5.15 ± 0.84	5.35 ± 0.90	4.97 ± 0.81	1.103, 0.003	1.181, 0.005	1.226, 0.005	1.139, 0.23
	25.	PLAG1	5.87 ± 0.55	5.26 ± 0.57	4.74 ± 0.57	4.93 ± 0.64	4.55 ± 0.49	−1.116, 0.001	−1.110, 0.009	−1.067, 0.99	−0.866, 0.045
	26.	SCGB3A1	14.25 ± 0.40	13.93 ± 0.59	13.48 ± 0.78	13.89 ± 0.51	13.09 ± 0.85	−1.023, 0.025	−1.033, 0.092	−1.002, 0.99	−1.064, 0.006
	27.	LHX6	5.33 ± 0.35	5.80 ± 0.44	6.24 ± 0.57	6.28 ± 0.61	6.20 ± 0.59	1.087, 0.001	1.076, 0.016	1.084, 0.084	1.069, 0.25
	28.	LINC00942	3.57 ± 0.17	3.85 ± 0.69	4.45 ± 1.28	4.67 ± 1.53	4.24 ± 1.09	1.079, 0.073	1.156, 0.18	1.212, 0.008	1.101, 0.828
	29.	REEP1	9.40 ± 0.60	9.79 ± 0.55	9.81 ± 0.74	9.91 ± 0.91	9.71 ± 0.63	1.041, 0.013	1.002, 0.716	1.013, 0.99	−1.009, 0.99
	30.	C6orf164	5.53 ± 0.51	6.06 ± 0.56	6.08 ± 0.52	6.17 ± 0.53	5.99 ± 0.56	1.097, 0.001	1.003, 0.66	1.017, 0.99	−1.012, 0.99
	31.	LINC00589	5.15 ± 0.27	5.38 ± 0.32	5.32 ± 0.29	5.33 ± 0.16	5.30 ± 0.41	1.045, 0.001	−1.011, 0.848	−1.010, 0.99	−1.015, 0.99
	32.	JAKMIP3	4.50 ± 0.22	5.25 ± 0.61	6.18 ± 0.81	6.20 ± 0.92	6.16 ± 0.80	1.166, 0.001	1.177, 0.002	1.180, 0.001	1.174, 0.001
	33.	LINC00930	6.02 ± 0.36	6.90 ± 0.59	6.77 ± 0.71	6.87 ± 0.79	6.66 ± 0.69	1.146, 0.001	−1.019, 0.716	−1.004, 0.99	−1.035, 0.99
	34.	DNHD1	6.68 ± 0.50	7.15 ± 0.54	7.37 ± 0.83	7.47 ± 0.98	7.27 ± 0.75	1.070, 0.001	1.031, 0.358	1.044, 0.99	1.017, 0.99
	35.	TMCC3	3.80 ± 0.28	4.11 ± 0.48	4.48 ± 0.53	4.56 ± 0.62	4.40 ± 0.48	1.082, 0.001	1.090, 0.019	1.109, 0.12	1.071, 0.85
	36.	ADH7	8.08 ± 0.94	10.78 ± 0.72	10.87 ± 0.60	11.09 ± 0.59	10.66 ± 0.58	1.335, 0.001	1.008, 0.804	1.029, 0.99	−1.012, 0.99
	37.	PRKAR2B	7.35 ± 0.61	6.46 ± 0.74	5.74 ± 0.51	5.83 ± 0.46	5.64 ± 0.59	−1.137, 0.001	−1.125, 0.004	−1.107, 0.239	−1.145, 0.054
	38.	GAD1	4.87 ± 0.56	6.25 ± 0.99	7.44 ± 0.87	7.26 ± 1.01	7.62 ± 0.78	1.283, 0.001	1.190, 0.002	1.162, 0.068	1.219, 0.005
	39.	LOC338667	5.41 ± 0.34	5.20 ± 0.24	5.03 ± 0.22	5.04 ± 0.20	5.03 ± 0.26	−1.040, 0.002	−1.034, 0.071	−1.031, 0.99	−1.034, 0.99
	40.	CYB5A	4.73 ± 0.20	4.73 ± 0.18	4.61 ± 0.23	4.70 ± 0.19	4.53 ± 0.26	−1.000, 0.99	−1.026, 0.235	−1.006, 0.99	−1.043, 0.99
	41.	PIEZO2	7.34 ± 0.84	6.49 ± 0.87	5.71 ± 0.64	5.71 ± 0.79	5.71 ± 0.54	−1.132, 0.001	−1.137, 0.008	−1.136, 0.291	−1.135, 0.255
	42.	SLITRK6	8.05 ± 0.59	6.89 ± 0.84	6.01 ± 0.99	6.10 ± 1.31	5.93 ± 0.67	−1.169, 0.001	−1.146, 0.015	−1.129, 0.249	−1.161, 0.043
	43.	KCNA1	6.95 ± 1.04	5.79 ± 0.96	5.19 ± 0.72	5.27 ± 0.96	5.11 ± 0.47	−1.200, 0.001	−1.116, 0.031	−1.098, 0.99	−1.133, 0.658
	44.	LOC100507560	5.90 ± 0.71	5.45 ± 0.62	5.03 ± 0.65	5.01 ± 0.75	5.05 ± 0.63	−1.083, 0.006	−1.083, 0.041	−1.087, 0.99	−1.079, 0.99
**Age group**		**Gene Symbol**	**Healthy Non-Smoker (N = 8)**	**Healthy Smoker (N = 9)**	**COPD smoker (N = 11)**	**COPD stage I smoker (N = 4)**	**COPD stage II smoker (N = 7)**	**Fold Regulation**, **adjusted p-value (HS vs**. **HNS)**	**Fold Regulation**, **adjusted p-value (COPD vs**. **HS)**	**Fold Regulation**, **adjusted p-value (stage I vs**. **HS)**	**Fold Regulation**, **adjusted p-value (stage II vs**. **HS)**
≥50 years old	1.	PPP4R4	6.64 ± 0.64	6.05 ± 1.16	5.87 ± 0.63	6.20 ± 0.23	5.70 ± 0.75	−1.098, 0.99	−1.031, 0.191	1.024, 0.99	−1.062, 0.99
	2.	THSD4	7.29 ± 0.62	7.15 ± 0.33	6.99 ± 0.53	7.48 ± 0.33	6.72 ± 0.42	−1.019, 0.99	−1.023, 0.366	1.046, 0.99	−1.065, 0.663
	3.	NRG1	3.71 ± 0.18	4.02 ± 0.63	3.72 ± 0.22	3.80 ± 0.15	3.68 ± 0.25	1.084, 0.606	−1.081, 0.315	−1.059, 0.99	−1.095, 0.447
	4.	SCGB1A1	14.38 ± 0.13	14.38 ± 0.30	14.27 ± 0.13	14.23 ± 0.10	14.28 ± 0.16	1.000, 0.99	−1.008, 0.132	−1.010, 0.99	−1.007, 0.99
	5.	AHRR	3.79 ± 0.23	5.13 ± 0.74	5.19 ± 0.82	5.15 ± 0.96	5.21 ± 0.81	1.353, 0.012	1.012, 0.92	1.004, 0.99	1.016, 0.99
	6.	CYP1A1	4.64 ± 0.23	7.88 ± 1.63	7.55 ± 1.46	7.83 ± 1.26	7.40 ± 1.64	1.698, 0.001	−1.044, 0.763	−1.007, 0.99	−1.065, 0.99
	7.	CYP1B1	3.68 ± 0.43	9.76 ± 1.12	9.28 ± 1.15	9.55 ± 0.47	9.12 ± 1.43	2.650, 0.001	−1.052, 0.546	−1.022, 0.99	−1.069, 0.99
	8.	PRDM11	5.42 ± 0.18	5.32 ± 0.12	5.35 ± 0.26	5.41 ± 0.27	5.32 ± 0.27	−1.019, 0.99	1.006, 0.841	1.018, 0.99	−1.000, 0.99
	9.	CBR3	7.06 ± 0.44	8.60 ± 0.54	8.15 ± 0.63	8.17 ± 0.47	8.14 ± 0.74	1.218, 0.001	−1.055, 0.159	−1.052, 0.99	−1.056, 0.99
≥50 years old	10.	AKR1C1	6.20 ± 0.40	8.75 ± 0.90	7.97 ± 1.15	8.28 ± 1.14	7.80 ± 1.21	1.412, 0.001	−1.098, 0.159	−1.057, 0.99	−1.121, 0.596
	11.	AKR1C3	10.24 ± 0.28	12.18 ± 0.59	11.79 ± 0.63	11.92 ± 0.42	11.72 ± 0.75	1.190, 0.001	−1.033, 0.269	−1.022, 0.99	−1.040, 0.911
	12.	HTR2B	3.85 ± 0.21	4.17 ± 0.22	4.22 ± 0.28	4.05 ± 0.18	4.32 ± 0.28	1.083, 0.142	1.012, 0.99	−1.028, 0.99	1.036, 0.99
	13.	GRM1	3.75 ± 0.15	4.37 ± 0.54	3.98 ± 0.17	3.99 ± 0.17	3.97 ± 0.19	1.164, 0.008	−1.098, 0.056	−1.095, 0.328	−1.099, 0.141
	14.	CYP4Z1	6.58 ± 0.45	6.02 ± 0.45	5.95 ± 0.42	5.92 ± 0.53	5.96 ± 0.38	−1.094, 0.20	−1.012, 0.688	−1.017, 0.99	−1.009, 0.99
	15.	UCHL1	5.18 ± 0.35	9.51 ± 1.42	8.56 ± 1.54	8.03 ± 1.04	8.89 ± 1.74	1.836, 0.001	−1.111, 0.269	−1.185, 0.467	−1.070, 0.99
	16.	CABYR	4.89 ± 0.32	7.69 ± 1.35	7.13 ± 0.99	7.36 ± 0.83	7.00 ± 1.11	1.575, 0.001	−1.079, 0.228	−1.045, 0.99	−1.099, 0.99
	17.	GPRC5A	7.60 ± 0.45	7.60 ± 0.20	7.88 ± 0.61	7.79 ± 0.84	7.93 ± 0.50	1.000, 0.99	1.037, 0.108	1.024, 0.99	1.042, 0.99
	18.	CCDC37	9.33 ± 0.52	9.48 ± 0.36	9.02 ± 0.50	9.00 ± 0.86	9.04 ± 0.22	1.016, 0.99	−1.051, 0.044	−1.054, 0.982	−1.049, 0.701
	19.	GLI3	7.39 ± 0.36	6.91 ± 0.25	6.73 ± 0.25	6.81 ± 0.39	6.68 ± 0.15	−1.069, 0.044	−1.027, 0.269	−1.015, 0.99	−1.034, 0.99
	20.	ABCC3	6.92 ± 0.59	7.66 ± 0.52	7.31 ± 0.75	7.16 ± 0.48	7.40 ± 0.89	1.106, 0.379	−1.048, 0.315	−1.070, 0.99	−1.034, 0.99
	21.	SAMD5	3.77 ± 0.15	4.08 ± 0.33	3.75 ± 0.24	3.85 ± 0.28	3.70 ± 0.22	1.082, 0.183	−1.088, 0.044	−1.059, 0.953	−1.104, 0.06
	22.	RASSF10	7.48 ± 0.40	6.90 ± 0.79	6.81 ± 0.46	6.97 ± 0.17	6.72 ± 0.56	−1.083, 0.534	−1.013, 0.366	1.010, 0.99	−1.027, 0.99
	23.	USP27X	7.32 ± 0.36	6.88 ± 0.39	6.77 ± 0.27	6.67 ± 0.19	6.82 ± 0.31	−1.064, 0.168	−1.016, 0.482	−1.031, 0.99	−1.008, 0.99
	24.	NR0B1	3.84 ± 0.18	4.68 ± 0.72	4.43 ± 0.52	4.35 ± 0.47	4.48 ± 0.57	1.217, 0.041	−1.056, 0.421	−1.076, 0.99	−1.044, 0.99
	25.	PLAG1	5.35 ± 0.50	5.23 ± 0.41	4.88 ± 0.53	4.98 ± 0.50	4.82 ± 0.58	−1.023, 0.99	−1.072, 0.159	−1.050, 0.99	−1.085, 0.99
	26.	SCGB3A1	13.96 ± 0.39	13.88 ± 0.75	12.87 ± 0.79	13.10 ± 1.20	12.73 ± 0.49	−1.006, 0.99	−1.078, 0.044	−1.059, 0.642	−1.090, 0.042
	27.	LHX6	5.55 ± 0.26	5.89 ± 0.46	6.01 ± 0.46	5.91 ± 0.33	6.07 ± 0.53	1.062, 0.863	1.020, 0.269	1.003, 0.99	1.030, 0.99
	28.	LINC00942	3.64 ± 0.20	4.02 ± 0.93	3.83 ± 0.44	3.77 ± 0.27	3.86 ± 0.53	1.105, 0.987	−1.050, 0.841	−1.066, 0.99	−1.042, 0.99
	29.	REEP1	8.96 ± 0.75	10.11 ± 0.61	9.43 ± 0.78	8.77 ± 0.41	9.84 ± 0.66	1.129, 0.028	−1.072, 0.088	−1.153, 0.024	−1.028, 0.99
	30.	C6orf164	5.27 ± 0.44	6.38 ± 0.51	6.33 ± 0.68	6.18 ± 0.35	6.42 ± 0.82	1.212, 0.016	−1.008, 0.841	−1.033, 0.99	1.006, 0.99
	31.	LINC00589	5.14 ± 0.20	5.42 ± 0.33	5.47 ± 0.32	5.35 ± 0.20	5.55 ± 0.36	1.053, 0.609	1.009, 0.763	−1.012, 0.99	1.024, 0.99
	32.	JAKMIP3	4.55 ± 0.17	5.52 ± 0.60	5.26 ± 0.63	5.16 ± 0.78	5.31 ± 0.58	1.214, 0.022	−1.049, 0.482	−1.069, 0.99	−1.039, 0.99
	33.	LINC00930	5.98 ± 0.79	7.01 ± 0.41	6.34 ± 0.59	6.16 ± 0.76	6.44 ± 0.52	1.172, 0.061	−1.106, 0.044	−1.137, 0.348	−1.088, 0.767
	34.	DNHD1	6.42 ± 0.27	7.07 ± 0.51	6.94 ± 0.61	6.77 ± 0.80	7.04 ± 0.53	1.100, 0.202	−1.019, 0.92	−1.045, 0.99	−1.004, 0.99
	35.	TMCC3	4.03 ± 0.80	4.02 ± 0.55	4.43 ± 0.35	4.42 ± 0.23	4.44 ± 0.42	−1.002, 0.99	1.102, 0.035	1.098, 0.99	1.105, 0.99
	36.	ADH7	7.63 ± 0.89	11.08 ± 0.44	10.54 ± 0.63	10.48 ± 0.29	10.58 ± 0.78	1.453, 0.001	−1.051, 0.044	−1.058, 0.99	−1.048, 0.99
	37.	PRKAR2B	7.38 ± 0.42	6.38 ± 0.55	5.97 ± 0.67	5.96 ± 0.93	5.97 ± 0.55	−1.158, 0.04	−1.069, 0.228	−1.070, 0.99	−1.068, 0.99
	38.	GAD1	5.22 ± 0.87	6.89 ± 1.08	7.09 ± 0.86	6.85 ± 1.32	7.23 ± 0.57	1.321, 0.025	1.029, 0.688	−1.007, 0.99	1.049, 0.99
	39.	LOC338667	5.32 ± 0.20	5.14 ± 0.22	5.05 ± 0.29	5.02 ± 0.17	5.06 ± 0.35	−1.035, 0.99	−1.018, 0.315	−1.024, 0.99	−1.015, 0.99
	40.	CYB5A	4.70 ± 0.17	4.58 ± 0.18	4.45 ± 0.23	4.53 ± 0.27	4.41 ± 0.21	−1.026, 0.99	−1.029, 0.228	−1.012, 0.99	−1.040, 0.81
	41.	PIEZO2	6.64 ± 0.77	6.52 ± 0.33	6.05 ± 0.61	5.86 ± 0.74	6.16 ± 0.57	−1.019, 0.99	−1.078, 0.159	−1.111, 0.81	−1.058, 0.99
	42.	SLITRK6	7.50 ± 0.62	6.67 ± 0.96	6.46 ± 0.51	6.60 ± 0.69	6.38 ± 0.42	−1.125, 0.298	−1.033, 0.482	−1.010, 0.99	−1.046, 0.99
	43.	KCNA1	6.40 ± 1.00	5.83 ± 0.82	5.20 ± 0.85	5.79 ± 0.86	4.89 ± 0.69	−1.098, 0.99	−1.121, 0.159	−1.007, 0.99	−1.193, 0.356
	44.	LOC100507560	5.59 ± 0.52	5.20 ± 0.52	4.82 ± 0.22	4.81 ± 0.18	4.83 ± 0.25	−1.075, 0.638	−1.079, 0.088	−1.081, 0.85	−1.077, 0.655

The Adj. P is based on the marginally adjusted 𝑝 values by the Benjamini-Hochberg-FDR correction at α = 0.05; Median ± Interquartile range.

We then investigated the differential regulation of these 44 candidate genes in HNS, HS, COPD patients, and Stage I and II patients over 50 years (Table 7; ≥50 years). In this age group, gene expression was not significantly different. This was surprising, as the symptoms of COPD worsen with age and one would expect associated gene regulation to become more dysregulated. Specifically, a comparison of HS to HNS in subjects over 50 years revealed that expression of 16/44 (36.4%) of the candidate genes and 5/17 (29.4%) 17 novel genes were significantly different (Table 7; ≥50 years; HS v HNS). Investigation of gene expression in COPD versus HS in subjects over 50 years revealed that expression of 3/44 (6.8%) of the 44 candidate genes and 1/17 (5.9%) of the novel genes were significantly different (Table 7; ≥ 50 years; COPD v HS). Subsequently, we investigated the differential gene expression in Stage I and II COPD patients over 50 years and determined that 1/44% (2.3%; Stage I) and 0/44 (0%; Stage II), respectively, were significantly different in patients ≤50 years. In our cohort of 17 novel genes, we determined that expression of only 1/17% (5.9%; Stage I) and 0/17 (0%; Stage II) genes, respectively, were significantly different in patients ≤50 years (Table 7; ≥50 years; Stage I and Stage II v HS). Furthermore, in COPD patients over 50 years, no genes were significantly different across all four analysis groups (i.e. HS v HNS; COPD v HS; Stage I v HS and Stage II v HS).

### Investigation of the effect of cigarette pack number per year on differential gene expression in HS and COPD (Stage I and II) patients

Here, we also investigated the effect of cigarette pack number per year (i.e. < or ≥50 cigarette packs/year) on the gene expression in HS, COPD patients, and Stage I and II patients. We analyzed the differential regulation of the 44 candidate genes in HS, COPD patients, and Stage I and II patients who consumed less than 50 packs/year (Table 8; <50 packs/year). In this age group, gene expression was not significantly different. Investigation of gene expression in COPD versus HS in subjects who consumed less than 50 packs/year revealed that expression of 4/44 (9.1%) candidate genes and 2/17 (11.8%) novel genes were significantly different (Table 8; <50 packs/year; COPD v HS). Subsequently, we studied the differential gene expression in Stage II COPD patients who consumed ≥50 packs/year and determined that 4/44 (9.1%) candidate genes, were significantly different in patients ≤50 years. In our cohort of 17 novel genes, we determined that the expression of only 1/17 (5.9%) of Stage II genes was significantly different in patients who consumed ≥50 packs/year compared to HS (Table 8; <50 packs/year; Stage I and Stage II v HS). Furthermore, a certain number of candidate genes were significantly different in subjects who consumed ≥50 packs/year, across in both analysis groups, which included SAMD5, PLAG1 and the novel gene, SLITRK6.

**Table 8:** Comparison of relative expression of 44 candidate genes between healthy controls (smokers and non-smokers) and COPD smoker patients (stage I and stage II) in number of pack of cigarette per year, separately.

Smoking group		Gene Symbol	Healthy Smoker (N = 37)	COPD smoker (N = 6)	COPD stage I smoker (N = 0)	COPD stage II smoker (N = 6)	Fold Regulation, adjusted p-value (COPD vs. HS)	Fold Regulation, adjusted p-value (stage I vs. HS)	Fold Regulation, adjusted p-value (stage II vs. HS)
<50 packs per year	1.	PPP4R4	6.69 ± 0.53	5.81 ± 0.10	—	5.81 ± 0.10	−1.153, 0.074	—	−1.153, 0.074
	2.	THSD4	7.28 ± 0.38	6.99 ± 0.26	—	6.99 ± 0.26	−1.042, 0.369	—	−1.042, 0.369
	3.	NRG1	3.97 ± 0.52	3.66 ± 0.01	—	3.66 ± 0.01	−1.086, 0.428	—	−1.086, 0.428
	4.	SCGB1A1	14.31 ± 0.36	14.40 ± 0.23	—	14.40 ± 0.23	1.007, 0.762	—	1.007, 0.762
	5.	AHRR	5.01 ± 0.44	5.74 ± 1.43	—	5.74 ± 1.43	1.145, 0.572	—	1.145, 0.572
	6.	CYP1A1	5.93 ± 1.39	7.84 ± 1.61	—	7.84 ± 1.61	1.321, 0.18	—	1.321, 0.18
	7.	CYP1B1	8.60 ± 1.25	10.10 ± 0.43	—	10.10 ± 0.43	1.175, 0.189	—	1.175, 0.189
	8.	PRDM11	5.41 ± 0.21	5.41 ± 0.29	—	5.41 ± 0.29	1.001, 0.971	—	1.001, 0.971
	9.	CBR3	8.18 ± 0.48	8.89 ± 0.59	—	8.89 ± 0.59	1.086, 0.159	—	1.086, 0.159
	10.	AKR1C1	9.19 ± 0.39	8.91 ± 0.86	—	8.91 ± 0.86	−1.031, 0.737	—	−1.031, 0.737
	11.	AKR1C3	12.26 ± 0.38	12.45 ± 0.12	—	12.45 ± 0.12	1.015, 0.555	—	1.015, 0.555
	12.	HTR2B	4.04 ± 0.27	4.33 ± 0.36	—	4.33 ± 0.36	1.072, 0.282	—	1.072, 0.282
	13.	GRM1	4.30 ± 0.62	4.14 ± 0.25	—	4.14 ± 0.25	−1.038, 0.708	—	−1.038, 0.708
	14.	CYP4Z1	6.13 ± 0.34	6.10 ± 0.05	—	6.10 ± 0.05	−1.006, 0.876	—	−1.006, 0.876
	15.	UCHL1	10.40 ± 0.84	10.34 ± 0.52	—	10.34 ± 0.52	−1.006, 0.908	—	−1.006, 0.908
	16.	CABYR	8.02 ± 0.55	8.08 ± 1.23	—	8.08 ± 1.23	1.007, 0.939	—	1.007, 0.939
	17.	GPRC5A	7.21 ± 0.38	7.80 ± 0.07	—	7.80 ± 0.07	1.082, 0.094	—	1.082, 0.094
	18.	CCDC37	9.24 ± 0.23	8.88 ± 0.01	—	8.88 ± 0.01	−1.040, 0.086	—	−1.040, 0.086
	19.	GLI3	6.57 ± 0.44	6.72 ± 0.03	—	6.72 ± 0.03	1.023, 0.685	—	1.023, 0.685
	20.	ABCC3	8.13 ± 0.40	7.80 ± 0.39	—	7.80 ± 0.39	−1.042, 0.371	—	−1.042, 0.371
	21.	SAMD5	4.31 ± 0.34	3.44 ± 0.08	—	3.44 ± 0.08	−1.252, 0.003	—	−1.252, 0.003
	22.	RASSF10	7.33 ± 0.37	6.52 ± 1.20	—	6.52 ± 1.20	−1.125, 0.529	—	−1.125, 0.529
	23.	USP27X	7.12 ± 0.44	6.61 ± 0.08	—	6.61 ± 0.08	−1.077, 0.18	—	−1.077, 0.18
	24.	NR0B1	4.41 ± 0.43	5.12 ± 0.70	—	5.12 ± 0.70	1.160, 0.145	—	1.160, 0.145
	25.	PLAG1	5.37 ± 0.15	4.56 ± 0.34	—	4.56 ± 0.34	−1.178, 0.005	—	−1.178, 0.005
	26.	SCGB3A1	13.83 ± 0.81	13.18 ± 0.05	—	13.18 ± 0.05	−1.049, 0.319	—	−1.049, 0.319
	27.	LHX6	5.86 ± 0.34	6.27 ± 0.70	—	6.27 ± 0.70	1.070, 0.549	—	1.070, 0.549
	28.	LINC00942	4.20 ± 1.01	3.86 ± 0.22	—	3.86 ± 0.22	−1.088, 0.417	—	−1.088, 0.417
	29.	REEP1	10.30 ± 0.30	10.13 ± 0.37	—	10.13 ± 0.37	−1.017, 0.629	—	−1.017, 0.629
	30.	C6orf164	6.39 ± 0.44	6.60 ± 0.37	—	6.60 ± 0.37	1.033, 0.587	—	1.033, 0.587
	31.	LINC00589	5.34 ± 0.11	5.55 ± 0.21	—	5.55 ± 0.21	1.040, 0.115	—	1.040, 0.115
	32.	JAKMIP3	5.44 ± 0.26	5.73 ± 0.11	—	5.73 ± 0.11	1.052, 0.222	—	1.052, 0.222
	33.	LINC00930	7.36 ± 0.41	6.71 ± 0.05	—	6.71 ± 0.05	−1.097, 0.022	—	−1.097, 0.022
	34.	DNHD1	7.08 ± 0.45	6.97 ± 0.47	—	6.97 ± 0.47	−1.016, 0.774	—	−1.016, 0.774
	35.	TMCC3	4.39 ± 0.64	4.74 ± 0.44	—	4.74 ± 0.44	1.081, 0.548	—	1.081, 0.548
	36.	ADH7	11.06 ± 0.20	11.17 ± 0.31	—	11.17 ± 0.31	1.010, 0.582	—	1.010, 0.582
	37.	PRKAR2B	6.48 ± 0.51	5.64 ± 0.57	—	5.64 ± 0.57	−1.149, 0.11	—	−1.149, 0.11
	38.	GAD1	7.65 ± 0.56	7.33 ± 0.04	—	7.33 ± 0.04	−1.044, 0.46	—	−1.044, 0.46
	39.	LOC338667	5.06 ± 0.25	4.83 ± 0.07	—	4.83 ± 0.07	−1.047, 0.276	—	−1.047, 0.276
	40.	CYB5A	4.67 ± 0.22	4.38 ± 0.02	—	4.38 ± 0.02	−1.066, 0.139	—	−1.066, 0.139
	41.	PIEZO2	6.46 ± 0.34	6.44 ± 0.32	—	6.44 ± 0.32	−1.003, 0.931	—	−1.003, 0.931
	42.	SLITRK6	7.17 ± 0.10	6.61 ± 0.05	—	6.61 ± 0.05	−1.085, 0.001	—	−1.085, 0.001
	43.	KCNA1	5.76 ± 0.52	5.45 ± 0.56	—	5.45 ± 0.56	−1.056, 0.517	—	−1.056, 0.517
	44.	LOC100507560	5.09 ± 0.46	4.90 ± 0.30	—	4.90 ± 0.30	−1.038, 0.607	—	−1.038, 0.607
**Smoking group**		**Gene Symbol**	**Healthy Smoker (N = 22)**	**COPD smoker (N = 15)**	**COPD stage I smoker (N = 9)**	**COPD stage II smoker (N = 6)**	**Fold Regulation**, **adjusted p-value (COPD vs**. **HS)**	**Fold Regulation**, **adjusted p-value (stage I vs**. **HS)**	**Fold Regulation**, **adjusted p-value (stage II vs**. **HS)**
≥50 packs per year	1.	PPP4R4	5.95 ± 0.89	5.49 ± 0.71	5.82 ± 0.43	5.23 ± 0.82	−1.084, 0.047	−1.023, 0.99	−1.138, 0.054
	2.	THSD4	7.34 ± 0.56	7.11 ± 0.54	7.51 ± 0.32	6.81 ± 0.50	−1.032, 0.178	1.022, 0.99	−1.078, 0.02
	3.	NRG1	4.06 ± 0.47	3.82 ± 0.41	3.97 ± 0.50	3.70 ± 0.28	−1.063, 0.014	−1.021, 0.99	−1.096, 0.03
	4.	SCGB1A1	14.37 ± 0.18	14.12 ± 0.33	14.20 ± 0.26	14.06 ± 0.37	−1.018, 0.001	−1.012, 0.135	−1.022, 0.001
	5.	AHRR	4.55 ± 0.67	5.18 ± 0.72	5.47 ± 0.86	4.96 ± 0.52	1.138, 0.001	1.203, 0.001	1.092, 0.169
	6.	CYP1A1	6.15 ± 1.89	7.78 ± 1.88	8.25 ± 1.49	7.42 ± 2.18	1.265, 0.003	1.342, 0.002	1.207, 0.059
	7.	CYP1B1	7.67 ± 2.10	9.43 ± 1.23	9.95 ± 0.63	9.03 ± 1.49	1.229, 0.006	1.298, 0.004	1.177, 0.15
	8.	PRDM11	5.35 ± 0.34	5.25 ± 0.29	5.32 ± 0.22	5.19 ± 0.34	−1.019, 0.239	−1.005, 0.99	−1.030, 0.99
	9.	CBR3	8.15 ± 0.70	8.33 ± 0.63	8.57 ± 0.46	8.14 ± 0.71	1.022, 0.383	1.052, 0.428	−1.001, 0.99
≥50 packs per year	10.	AKR1C1	8.51 ± 1.15	8.21 ± 1.25	8.36 ± 1.30	8.09 ± 1.27	−1.037, 0.475	−1.018, 0.99	−1.051, 0.99
	11.	AKR1C3	11.8 ± 20.73	11.88 ± 0.68	12.16 ± 0.49	11.66 ± 0.76	1.007, 0.968	1.029, 0.92	−1.014, 0.99
	12.	HTR2B	4.23 ± 0.31	4.24 ± 0.33	4.18 ± 0.43	4.29 ± 0.23	1.002, 0.884	−1.012, 0.99	1.013, 0.99
	13.	GRM1	4.03 ± 0.30	4.16 ± 0.59	4.20 ± 0.39	4.13 ± 0.74	1.032, 0.771	1.042, 0.811	1.026, 0.99
	14.	CYP4Z1	6.25 ± 0.60	5.92 ± 0.40	5.93 ± 0.48	5.91 ± 0.35	−1.056, 0.034	−1.055, 0.706	−1.059, 0.379
	15.	UCHL1	8.65 ± 1.48	9.28 ± 1.87	9.52 ± 1.61	9.09 ± 2.13	1.073, 0.11	1.100, 0.463	1.051, 0.9
	16.	CABYR	6.83 ± 1.24	7.40 ± 1.44	8.06 ± 1.18	6.91 ± 1.50	1.083, 0.157	1.180, 0.01	1.011, 0.99
	17.	GPRC5A	7.54 ± 0.43	8.05 ± 0.65	7.92 ± 0.75	8.16 ± 0.58	1.068, 0.003	1.051, 0.353	1.083, 0.008
	18.	CCDC37	9.37 ± 0.55	9.39 ± 0.56	9.40 ± 0.66	9.38 ± 0.51	1.002, 0.802	1.003, 0.99	1.002, 0.99
	19.	GLI3	6.76 ± 0.59	6.62 ± 0.41	6.73 ± 0.45	6.54 ± 0.37	−1.021, 0.25	−1.004, 0.99	−1.034, 0.946
	20.	ABCC3	7.86 ± 0.62	7.65 ± 0.82	7.79 ± 0.74	7.53 ± 0.90	−1.027, 0.428	−1.008, 0.99	−1.044 0.82
	21.	SAMD5	3.95 ± 0.26	3.97 ± 0.50	3.98 ± 0.29	3.96 ± 0.64	1.005, 0.53	1.007, 0.99	1.003, 0.99
	22.	RASSF10	7.04 ± 0.60	6.61 ± 0.52	6.79 ± 0.33	6.47 ± 0.62	−1.065, 0.002	−1.036, 0.99	−1.088, 0.018
	23.	USP27X	7.13 ± 0.40	6.65 ± 0.44	6.72 ± 0.29	6.60 ± 0.54	−1.072, 0.001	−1.062, 0.017	−1.081, 0.001
	24.	NR0B1	4.39 ± 0.80	4.77 ± 0.79	5.00 ± 0.87	4.59 ± 0.70	1.087, 0.032	1.139, 0.058	1.047, 0.99
	25.	PLAG1	5.24 ± 0.58	4.84 ± 0.57	4.98 ± 0.58	4.74 ± 0.58	−1.083, 0.012	−1.054, 0.99	−1.106, 0.07
	26.	SCGB3A1	13.93 ± 0.59	13.25 ± 0.79	13.81 ± 0.51	12.82 ± 0.71	−1.051, 0.002	−1.008, 0.99	−1.087, 0.001
	27.	LHX6	5.80 ± 0.45	6.09 ± 0.52	6.08 ± 0.54	6.10 ± 0.53	1.050, 0.088	1.048, 0.498	1.050, 0.271
	28.	LINC00942	3.84 ± 0.68	4.16 ± 1.06	4.31 ± 1.30	4.05 ± 0.87	1.083, 0.29	1.121, 0.093	1.053, 0.99
	29.	REEP1	9.78 ± 0.56	9.64 ± 0.73	9.55 ± 0.86	9.71 ± 0.65	−1.015, 0.484	−1.024, 0.99	−1.007, 0.529
	30.	C6orf164	6.07 ± 0.56	6.19 ± 0.64	6.21 ± 0.45	6.17 ± 0.78	1.020, 0.376	1.023, 0.99	1.016, 0.99
	31.	LINC00589	5.39 ± 0.33	5.39 ± 0.33	5.35 ± 0.18	5.42 ± 0.41	1.000, 0.843	−1.008, 0.99	1.006, 0.99
	32.	JAKMIP3	5.26 ± 0.63	5.74 ± 0.88	5.86 ± 0.94	5.64 ± 0.87	1.091, 0.036	1.113, 0.019	1.071, 0.233
	33.	LINC00930	6.87 ± 0.57	6.60 ± 0.65	6.73 ± 0.70	6.50 ± 0.63	−1.041, 0.204	−1.021, 0.99	−1.057, 0.29
	34.	DNHD1	7.15 ± 0.54	7.25 ± 0.70	7.36 ± 0.78	7.17 ± 0.65	1.014, 0.721	1.029, 0.99	1.003, 0.99
	35.	TMCC3	4.07 ± 0.47	4.43 ± 0.45	4.51 ± 0.50	4.36 ± 0.41	1.088, 0.001	1.107, 0.055	1.072, 0.348
	36.	ADH7	10.79 ± 0.72	10.65 ± 0.65	10.84 ± 0.59	10.50 ± 0.69	−1.013, 0.245	1.005, 0.99	−1.027, 0.99
	37.	PRKAR2B	6.45 ± 0.74	5.85 ± 0.61	5.83 ± 0.68	5.87 ± 0.59	−1.103, 0.003	−1.106, 0.082	−1.099, 0.062
	38.	GAD1	6.20 ± 0.96	7.39 ± 0.75	7.38 ± 0.82	7.40 ± 0.74	1.192, 0.001	1.190, 0.001	1.195, 0.002
	39.	LOC338667	5.20 ± 0.23	5.07 ± 0.26	5.05 ± 0.18	5.09 ± 0.31	−1.026, 0.056	−1.031, 0.865	−1.022, 0.99
	40.	CYB5A	4.72 ± 0.19	4.54 ± 0.25	4.63 ± 0.25	4.47 ± 0.25	−1.040, 0.012	−1.018, 0.99	−1.054, 0.004
	41.	PIEZO2	6.49 ± 0.86	5.87 ± 0.63	5.86 ± 0.72	5.88 ± 0.59	−1.106, 0.007	−1.107, 0.268	−1.104, 0.168
	42.	SLITRK6	6.83 ± 0.88	6.18 ± 0.83	6.28 ± 1.11	6.11 ± 0.58	−1.105, 0.011	−1.088, 0.443	−1.119, 0.03
	43.	KCNA1	5.80 ± 0.97	5.10 ± 0.76	5.39 ± 0.89	4.89 ± 0.58	−1.137, 0.007	−1.076, 0.99	−1.186, 0.032
	44.	LOC100507560	5.46 ± 0.61	4.93 ± 0.51	4.94 ± 0.59	4.92 ± 0.48	−1.108, 0.001	−1.104, 209	−1.108, 0.087

The Adj. P is based on the marginally adjusted 𝑝 values by the Benjamini-Hochberg-FDR correction at α = 0.05; Median ± Interquartile range.

In COPD patients who consumed ≥50 packs/year, we observed a significant change in gene expression in 22/44 (50%) candidate genes and 9/17 (52.9%) novel genes compared with HS (Table 8; ≥50 packs/year; COPD v HS). We also investigated differential gene expression in Stage I and II COPD patients who consumed ≥50 packs/year and determined that 7/44 (15.9%; Stage I) and 10/44 (22.7%; Stage II) candidate genes were significantly different compared to gene expression in HSs. In our cohort of 17 novel genes, we determined that gene expression in 2/17% (11.8%; Stage I) and 4/17% (23.5%; Stage II) was significantly different in COPD patients who consumed ≥50 packs/year (Table 8; ≥50 packs/year; Stage I and Stage II v HS). In addition, a certain number of the 44 candidate genes were significantly different across all four-analysis groups, which included USP27X, CYP1A1 and the novel genes, PRKAR2B and GAD1.

## Discussion

Chronic obstructive pulmonary disease (COPD) is a progressive inflammatory disease characterized by airway obstruction and is predicted to be among the first three causes of death worldwide^1,2^. A significant degree of clinical heterogeneity has been observed in COPD patients. In functional terms, all COPD patients experience a loss in lung function, as measured using FEV1 and FVC. However, these clinical parameters are not optimal and FEV1 has been shown to correlate weakly with clinical outcome and health status^19,20^. Currently, there is an unmet clinical need to identify novel biomarkers that will facilitate improved diagnosis and prognosis in COPD.

To date, COPD has been shown to develop in 30% of smokers, with smoking being one of the main risk factors associated with the development of COPD^3^. The aim of this project was to identify candidate novel biomarkers, which may provide future novel therapeutic targets, in order to facilitate the treatment of COPD using machine-based learning algorithms and penalized regression models. In this study, 59 healthy smokers, 53 healthy non-smokers and 21 COPD smokers (9 GOLD stage I and 12 GOLD stage II) were included (n = 133). 20,097 probes were generated from SAE microarray data obtained from these subjects previously^14^. Consequently, 44 candidate genes were identified to be associated with the occurrence or progression of COPD, or lung function. Of these 44 genes, 27 have been previously reported in the literature to be associated with COPD or lung function (FVC, FEV_1_ or the FEV_1_/FVC ratio). In this study, we also identified 17 genes not previously detected in COPD studies that may represent novel biomarkers in the diagnosis and prognosis of COPD. In our analyses among healthy non-smokers and healthy smokers and COPD patients (GOLD stage I and II), the most significantly regulated novel genes were: PRKAR2B, GAD1, LINC00930 and SLITRK6.

PRKAR2B is a protein kinase type II-beta regulatory subunit dependent on cAMP and encoded by the PRKAR2B gene in human^21^. In our overall analyses, expression of PRKAR2B was significantly downregulated in healthy smokers and in COPD patients (and in COPD stage II) compared to healthy non-smokers. Furthermore, in males, PRKAR2B expression was also significantly downregulated in healthy smokers and in COPD patients compared to healthy non-smokers. In females, these differences were less pronounced. In subjects less than 50 years, PRKAR2B expression was significantly downregulated in healthy smokers and in COPD patients compared to healthy non-smokers. In patients over 50 years, these differences were less pronounced. With regards to smoking exposure, COPD patients who smoked more than 50 packs per year had significantly lower PRKAR2B gene expression than healthy non-smokers. This decrease was not evident in COPD patients who smoked less than 50 cigarette packs per year. Thus, we hypothesise that PRKAR2B may represent a previously unknown factor both in pathogenesis of COPD and smoking exposure. PRKAR2B is an important protein kinase in cAMP signaling, and other researchers have demonstrated that cAMP is a protective factor in the lung and COPD. Furthermore, cAMP has been shown to attenuate pro-inflammatory responses whilst concomitantly increasing anti-inflammatory responses in a number of innate immune cells^22^. The reduced PRKAR2B gene expression observed in this study may reveal PRKAR2B as a novel target in the treatment of COPD.

In contrast, in this study we also observed a significant upregulation of the novel gene, GAD1, in healthy smokers and COPD patients (and in stage II) compared to healthy non-smokers. In male only subjects, this pattern was replicated. The increase in expression of GAD1 in healthy smokers and COPD patients compared to healthy non-smokers was marginally less significant than in male subjects, as expected. In subjects younger than 50 years, there was a more significant increase in GAD1 expression in healthy smokers and COPD patients compared to subjects over 50 years and also the non-smokers. Smoking exposure only significantly increased GAD1 levels in healthy smokers and COPD patients who smoked more than 50 packs per year. There were no significant changes in GAD1 expression in subjects who smoked less than 50 packs per year. Other studies have shown that levels of γ-aminobutyric acid (GABA) and glutamic acid decarboxylase 1 (GAD1), the enzyme that synthesizes GABA, are significantly increased in neoplastic tissues^23^. Furthermore, other researchers have shown that the GAD1 promoter is hypermethylated in a number of cancer cells. This effect was shown to lead to the production of high levels of GAD1, as opposed to gene silencing which one would expect. The GAD1 promoter contains a number of CpG island motifs which facilitate this hypermethylation. In this study, we hypothesise that the increased levels of GAD1 detected following smoking exposure and COPD could mean that GAD1 is an important target in the treatment of this smoking-related disease. Previous studies have demonstrated that patients with COPD are at an increased risk for both the development of primary lung cancer, as well as poor outcome after lung cancer diagnosis and treatment^24^. Targeting the knockdown of GAD1 in COPD may attenuate the increased risk of lung cancer in COPD patients.

LINC00930 was an additional novel gene detected in this study using machine-based learning. This is a “long intergenic non-protein coding (linc) RNA 930” (https://www.genecards.org/cgi-bin/carddisp.pl?gene=LINC00930). Interestingly, some other novel genes including LINC00942 and LINC00589 were also detected in this study. However, they were not as significantly regulated by smoking exposure or in COPD patients. In general, lincRNAs are found in between coding genes rather than antisense to them or within introns. Although the specific function of lincRNAs is not well known, they are thought to contribute to RNA stability in cells and hence gene expression. In our study, LINC009030 expression was significantly increased in healthy smokers overall, and in males and females, compared to healthy non-smokers. LINC009030 expression was significantly increased in smokers less than 50 years when only compared to non-smokers. In the over-50 age group, COPD patients had significantly less LINC009030 expression compared to healthy non-smokers. Regarding the smoking exposure, COPD patients and Stage II COPD patients who smoked less than 50 packs per year had significantly less LINC009030 expression compared to healthy non-smokers. In this study, LINC009030 was the only novel gene whose expression was significantly upregulated in smokers while significantly downregulated in COPD patients. Additional experimentation is required for elucidating the mechanism underlying this result in order to evaluate the therapeutic potential of targeting LINC009030 in smoking-related morbidities and in COPD patients.

SLITRK6 was the last novel gene, which we determined to be significantly regulated in smoking and in COPD in this study. SLITRK6 is a member of the SLITRK family of neuronal transmembrane proteins that was discovered as a bladder tumor antigen using suppressive subtractive hybridization^25^. Using immunohistochemistry, SLITRK6 has been shown to be extensively expressed in multiple epithelial tumors, including lung, bladder and breast cancer, as well as in glioblastoma^25^. In our study, we demonstrated that SLITRK6 was significantly downregulated in smokers and COPD patients (including stage II) compared to healthy non-smokers. Male smokers and COPD patients also had significantly lower SLITRK6 expression compared to non-smokers. In females, SLITRK6 expression was significantly less in smokers, but not COPD patients, compared to non-smokers. Smokers and COPD patients (including stage II) aged less than 50 years had significantly lower SLITRK6 expression compared to non-smokers. These effects were not evident in subjects over 50 years. Concerning the smoking exposure, COPD patients (including stage II) who smoked more than 50 packs per year had significantly lower SLITRK6 expression compared to non-smokers. This effect was also evident in COPD patients (including stage II) who smoked less than 50 packs per year. The highlight of this study was that the expression of the oncogenesis-promoting enzyme, GAD1, is significantly increased in response to smoking exposure and in COPD. Although SLITRK6 is considered a tumourogenesis promoting factor, it was significantly decreased in this study in responses to smoking exposure and in COPD. Here, we hypothesise that GAD1 may represent a better target in order to attenuate the incidence of cancer following COPD. However, further investigations are needed to explain the exact function of SLITRK6 in smoking-associated morbidities and in COPD.

More recently, machine-based learning algorithms have gained increasing attention in bioinformatics and biology research^26,27^. In contrast, regularization-based regression models (e.g. LASSO logistic regression) have already been used widely in microarray analysis^28^. Microarray analysis has a number of limitations including overfitting and multi-collinearity. In order to address these issues, regularization of parameters is required^29^. In this study, the area under the receiver operator characteristic curve (AUC), the sensitivity and specificity, and the misclassification error rate were quantitated for machine-based learning algorithm and penalty-based statistical method used. In this study based on repeated 5-CV, the elastic-net, random forest, and LASSO regularized logistic regression models were found to perform better than the naive Bayes, ridge, gradient boosting machines, adaptive boosting classification trees, extension of LASSO, artificial neural network, support vector machines, and decision tree models, respectively. Elastic-net regularization produced a sparse model with good prediction accuracy and good grouping-capability. This result is in keeping with those from previous studies, which demonstrated that elastic-net frequently performs better than ridge and LASSO for model selection consistency and prediction accuracy in microarray datasets^28,30,31^. Therefore, the results of this study are in agreement with those from previous ones.

In summary, we employed machine-based learning algorithms and penalized regression models in order to identify 44 candidate genes, whose expression was significantly regulated by smoking exposure and/or COPD. We also identified 17 novel genes which were not previously determined to be associated with smoking exposure or COPD. We determined that four of these novel genes, namely PRKARB2, GAD1, LINC00930 and SLITKR6, were the most significantly regulated by smoking exposure or in COPD. We also determined that elastic-net logistic regression in our dataset had a higher accuracy rate compared to the other algorithms. Therefore, in microarray data, elastic-net logistic regression may provide a useful methodology for future studies in the discovery of novel diagnostic- and prognostic-biomarkers, and novel therapeutic targets in the treatment of COPD and other smoking-related diseases.

The strengths of this study include the use of modern and accepted computational methods, the address of potential sources of bias, validation of all of the results by literature review, the use of appropriate cross-validation method (repeated 5-CV), enrichment analysis and the use of STRING networks. The main limitations of the study are the small sample sizes and that this is a case-control study only. Specifically, this study does not establish the respective associations between the 44 candidate genes and any COPD outcomes (e.g. lung function changes, exacerbations or mortality). Therefore, future studies by us will investigate and validate the candidacy of our 44 novel genes as novel therapeutic targets in COPD using larger patient cohorts. Furthermore, these studies, will investigate the association between these 44 novel genes and the change in lung function over time (FEV_1_ or FEV_1_/FVC ratio), incidence of exacerbations and mortality in COPD patients.

## Methods

### Study subjects and dataset

In this study, 59 healthy smokers, 53 healthy non-smokers and 21 COPD smokers (9 of stage I, GOLD I and 12 of stage II, GOLD II) were included (Total: n = 133). Subjects were predominantly male (n = 95; 71.4%) and Caucasian (n = 48; 36.1%; Table 1). Pulmonary function tests from COPD patients revealed that forced vital capacity (FVC), forced expiratory volume 1 (FEV1) and the FEV1/FVC ratio were significantly lower in these patients compared with healthy smokers and healthy non-smokers (Table 1). From these subjects, the raw data of gene expression architecture in the small airway epithelium (SAE) cells of COPD was used^14^. 20,097 probes from 133 subjects were generated. Genome-wide gene expression analysis (GWAS) was performed using HG-U133 Plus 2.0 array (Affymetrix, Santa Clara, CA)^14^. Overall microarray quality was verified by the criteria: (1) 3′/5′ ratio for GAPDH ≤3; and (2) scaling factor ≤10.0. The captured image data from the HG-U133 Plus 2.0 arrays was processed using MAS5 algorithm. The data was normalized using GeneSpring version 7.3.1 (Agilent technologies, Palo Alto, CA). See Supplemental Methods for further details. The raw data is available at the Gene Expression Omnibus (GEO) site (http://www.ncbi.nlm.nih.gov/geo/), accession number for this dataset is GSE20257.

### Gene expression analysis

Raw data (.CEL format) files were qualified, normalized, statistical comparison, removing batch effects and other unwanted variation were performed by “Affy,” “Limma” and “SVA” R packages, respectively. The cutoff of false discovery rate and fold-change for differentially expressed genes was considered at level of 0.10, and more than 2, respectively.

### Module identification

Sample progression discovery (SPD) as a novel unsupervised computational approach to identify patterns of biological progression underlying microarray gene expression data. SPD assumes that individual samples of a microarray dataset are related by an unknown biological process, and that each sample represents one unknown point along the progression of that process. SPD aims to organize the samples in a manner that reveals the underlying progression and to simultaneously identify subsets of genes that are responsible for that progression. This method does not depend on prior knowledge and only uses gene expression information^32^. In this method, divisive/consensus k-means as a clustering gene algorithm was used (200 iterations in each consensus k-means partitioning, and 0.7 threshold for module coherence). Also, least number of genes in each modules was 10. SPD analysis was done by MATLAB 7 software.

### Machine learning (ML) algorithms

Various ML algorithms including AdaBoost classification trees, decision tree, Gradient Boosting machines, Naive Bayes, neural network, random forest, support vector machine were performed in order to find genes associated with occurrence and progression of COPD, and the best ML method which has best accuracy and performance to predict COPD. All ML methods were applied using “adabag”, “CART”, “gbm”, “naivebayes”, “neuralnet”, “‘randomForest”, and “e1071” R packages.

### Adaptive LASSO, elastic-net, and ridge logistic regression

The ridge regression uses an L_2_ penalty to regularize parameters, all of the estimated coefficients are nonzero, and hence no gene selection is performed. But, LASSO regression use the L_1_ penalty instead, and hence provide automatic gene selection. In other hand, ridge penalty tends to shrink the coefficients of correlated variables toward each other, good for multi-collinearity, grouped selection. But, the lasso penalty is somewhat indifferent to the choice among a set of strong but correlated variables. Therefore, LASSO is good for simultaneous estimation and eliminating trivial genes but not good for grouped selection. Elastic-net is introduced as a compromise between these two techniques, and has a penalty which is a mix of L_1_ and L_2_ penalty, combine strength between ridge and lasso^33^. In adaptive LASSO regression where adaptive weights, inverse absolute value of LASSO coefficient was used for each variable as its weight in adaptive LASSO, are used for penalizing different coefficients in the L_1_ penalty. Similar to the lasso, the adaptive lasso is shown to be near-minimax optimal. Unlike to the LASSO, the adaptive LASSO is consistent for gene selection^34^. The mentioned penalized logistics regression methods were done by “glmnet” R package (https://cran.r-project.org/package=glmnet). In Table 9, the summary of each machine-learning and penalized statistical methods with some of advantages and limitations were mentioned.

**Table 9:** Gene selection methods: Definitions, acronyms and main advantages and limitations.

Gene Selection Method Name	Gene Selection Method Acronym	Main Advantages	Main Limitations
Least Absolute Shrinkage and Selection Operator	LASSO	(1) Smaller mean squared error (MSE) than conventional methods; (2) It is good for simultaneous estimation and eliminating trivial genes; (3) Coefficients being easy to implement is another of the merits.	(1) It is not good for grouped selection; (2) For highly correlated variables, conventional methods have predictive performance empirically observed to be better than LASSO; (3) This method has shown to not always provide consistent variable selection; (4) Its estimators are biased always; (5) Its efficiency depends greatly on the number of dimension of genes.
Adaptive Least Absolute Shrinkage and Selection Operator	Adapt. LASSO	(1) This method has all of advantages of the LASSO. (2) This method uses adaptive weights to penalize coefficients differently; (3) Adaptive LASSO provides a more consistent solution than LASSO.	(1) It is not good for grouped selection; (2) For highly correlated variables, conventional methods have predictive performance empirically observed to be better than adapt. LASSO; (3) Its estimators are biased always; (4) Its efficiency depends greatly on the number of dimension of genes.
Elastic net regularization	Elastic net	(1) This method selects groups of correlated variables together, shares nice properties of both the LASSO and ridge; (2) It can be considered for situations with p > n, it allows the number of selected features to exceed the sample size; (3) This method has predictive performance better than LASSO and ridge.	(1) It can only apply to two-class feature selection problems, it cannot resolve multi-class feature selection problems directly; (2) Its estimators aren’t robust against outliers
Ridge Logistic Regression	Ridge	(1) It handles the multi-collinearity problem (2) Ridge regression can reduce the variance (with an increasing bias); (3) Can improve predictive performance than ordinary least square approach.	(1) It is not able to shrink coefficients to exactly zero; (2) It cannot perform variable selection; it includes all of predictors (e.g. genes) in the final model; (3) It cannot handles the overfitting problem.
Support Vector Machines	SVM	(1) It has a regularization parameter for avoiding overfitting; (2) It uses the kernel trick; (3) It is defined by a convex optimization problem (no local optimization); (4) It is a powerful classifier that works well on a wide range of classification problems, in other words, it is very good when we have no idea on the data; (5) It can apply for high dimensional and not linearly separable situations.	(1) Choosing a good kernel function is not easy; (2) It has several key parameters that need to be set correctly to achieve the best classification results for any given problem; (3) Long training time for large datasets and large amount of training data; it was computationally intensive, especially the grid search for tuning its parameters; (4) Difficult to understand and interpret the final model, variable weights and individual impact.
Gradient Boosting Machines (stochastic)	GBM	(1) It can apply for high dimensional situations; (2) It works well in the situation with a lot of main and interaction parameters; (3) It can automatically select variables; (4) It is robust to outliers and missing data; (5) It can handle the numerous correlated and irrelevant variables problems; (6) It is an ensemble learning.	(1) Long training time for large datasets; (2) Difficult to understand and interpret the model; (3) Prone to overfitting.
Naive Bayes	NB	(1) It is easy to implement as a single learning; (2) If the its conditional independence assumption actually holds, a Naive Bayes classifier will converge quicker than discriminative models (e.g. logistic regression); (3) It needs less training data than other algorithms;	(1) Class conditional independence assumption for all of variables (e.g. genes); (2) It is defined by a local optimization problem.
Random Forest	RF	(1) It can apply for high dimensional situations; (2) It is robust to outliers and missing data; (3) It has less variance than a single decision tree; (4) Training each tree perform independently.	(1) It is complex; (2) It requires more computational resources and are also less intuitive; (3) Its prediction process using random forests is time-consuming than decision trees; (4) It assumes that model errors are uncorrelated and uniform.
Artificial Neural Network	ANN	(1) It is easy to implement; (2) It can approximate any function between the independent and dependent variables; (3) It handles all possible interactions between the dependent variables; (4) It does not require any assumptions, in other words, it is very good when we have no idea on the data.	(1) It solved for local optimization; (2) Parameters are hard to interpret; (3) Long training time for large neural networks.
Decision Trees	RT	(1) Easy to interpret and explain as a single learning; (2) It is very fast; (3) Its estimators are robust against outliers; (4) Can be combined with other decision techniques; (5) It handles missing values and filling them in with the most probable value.	(1) Prone to overfitting; (2) Instability; (3) This method has predictive performance worse than random forest; (4) It solved for local optimization.
AdaBoost Classification Trees (Adaptive Boosting)	ABCT	(1) It can be less susceptible to the overfitting problem than most learning algorithms; (2) It combines a set of weak learners in order to form a strong classifier and selection of weak classifier is easy; (3) It is a machine learning meta-algorithm.	(1) It can be sensitive to noisy data and outliers; (2) Requirement of a large amount of training data and long training time.

### Gene set enrichment analysis

Gene set enrichment analysis is a method to identify classes of genes that are over-represented in a large set of genes and may have an association with disease phenotypes (e.g. occurrence of COPD). The Comprehensive gene set enrichment analysis web server 2016 update called “Enrichr” was applied^35^.

### Cross-validation, stability and accuracy

K-fold cross-validation scheme (k-cv) is a very commonly employed technique used to evaluate classifier performance. K-CV estimation of the error is the average value of the errors committed in each fold. Thus, the K-CV error estimator depends on two factors: the training set and the partition into folds. Sensitivity analysis was performed to changes in the training set and sensitivity to changes in the folds^36^. The bootstrap (or subsampling) is another way to bring down the high variability of cross-validation, to aims stability selection^37^. Repeated cross-validation is a good strategy for (a) optimizing the complexity of regression models and (b) for a realistic estimation of prediction errors when the model is applied to new cases^38,39^. In the present study, the algorithms split the data set by using repeated random 100 times sub-sampling in 5-fold cross-validation, permuting the sample labels every time. Cross-validated performance was summarized by observed sensitivity and specificity, and misclassification error rate. Furthermore, the area under the Receiver Operator Characteristic (ROC) curve (AUC), was used to calculate of classifiers performance^40,41^. Also, in order to assessing literature validation for any results, literature mining was used in the PubMed databank. Interactive cluster heatmaps was applied by “heatmaply” R package (https://cran.r-project.org/web/packages/heatmaply)^42^. A heatmap is a popular graphical method for visualizing high-dimensional data. A static heatmap, as an interactive heatmaps, was used to represent biological data, in which colors are used to represent the values (importance index) in a matrix where columns and rows are the machine learning and statistical methods (instances) and genes selected (attributes), respectively. Rows and columns are sorted using a hierarchical clustering technique^43^. The study’s follow chart is shown in Fig. 1.

## Electronic supplementary material

Dataset 1
